# Insights From a New 1‐ha Permanent Forest Plot Reveal Differences Between Habitat Type and Similarities Between Forest Type in the Southwestern Amazon

**DOI:** 10.1002/ece3.71476

**Published:** 2025-05-22

**Authors:** Riley P. Fortier, Thalia Corahua‐Espinoza, Varun Swamy, Kenneth J. Feeley, Geoffrey R. Gallice

**Affiliations:** ^1^ Department of Biology University of Miami Coral Gables Florida USA; ^2^ Alliance for a Sustainable Amazon Potomac Maryland USA; ^3^ Universidad Nacional Amazónica de Madre de Dios Puerto Maldonado Madre de Dios Peru; ^4^ Sabin Center for Environment and Sustainability Wake Forest University Winston‐Salem North Carolina USA; ^5^ Fairchild Tropical Botanic Garden Coral Gables Florida USA; ^6^ Department of Engineering Pontifical Catholic University of Peru Lima Peru; ^7^ Department of Natural History Florida Museum of Natural History, University of Florida Gainesville Florida USA

**Keywords:** Brazil nut, disturbed forest, floodplain, Madre de Dios, Tambopata, terra firme, tropical forest

## Abstract

The southwestern Amazon is a biodiversity hotspot home to some of the oldest permanent forest dynamics plots in the basin. Despite the region's abundance of plots, we still know relatively little about how tree diversity and composition change across the region's precipitation gradient, between habitat types, and how disturbed and managed forests compare to protected, old‐growth forests since the majority of forest plots are located in protected forests. In this study, we first described a new 1‐ha permanent forest dynamics plot at the confluence of agricultural land and managed Brazil nut forest. We then compared the plot to others in the region to evaluate the relationship between precipitation and plot diversity, compositional differences between floodplain and terra firme forest, and differences in forest dynamics between our disturbed forest plot and old‐growth forest plots. Contrary to large‐scale patterns in tree diversity, we found no relationship between precipitation and tree alpha diversity for plots in the southwestern Amazon. There were, however, clear compositional differences between floodplain and terra firme forests. Annual change in the aboveground biomass of the new plot was higher than in other plots in the region. Similarly, annual rates of mortality and recruitment were lower and higher, respectively, in the new plot compared to the other plots. The floristic and structural similarities between plots in disturbed or managed forests and plots in old‐growth forests indicate a high resilience of tropical forests to low‐intensity disturbances. Our findings thus provide evidence that low‐intensity logging and low‐impact Brazil nut harvesting in the southwest Amazon do not significantly alter forest structure and composition in the medium to long term. Our new plot bolsters the representation of disturbed and managed forests in plot databases and will be an important resource for future studies of large‐scale patterns of forest diversity, structure, and dynamics.

## Graphical abstract

Southwestern Amazon forests are highly diverse, but information on how tree diversity and composition changes between habitat and forest types remains scarce. We conducted floristic analyses to investigate these changes across a precipitation gradient, between habitat types, and between disturbed and old‐growth forest.

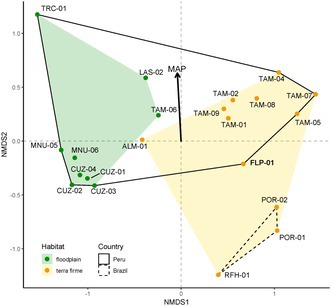

## 1 Introduction

The Amazon rainforest is the world's most diverse terrestrial ecosystem (ter Steege et al. 2020). The Peruvian department of Madre de Dios (MDD), located in the southwestern Amazon, is one of the most diverse regions in the entire basin and is Peru's self‐proclaimed “capital of biodiversity” (Peruvian Law No. 26311). The two dominant forested habitats in MDD are seasonally inundated floodplain forests and “terra firme” forests on raised terraces that are never inundated (Goulding et al. 2003). Our scientific knowledge of the forests of MDD is largely the result of the installation and continued monitoring of permanent forest dynamics plots. Despite the fact that MDD is home to some of the oldest plots in the Amazon (Gentry 1988; ForestPlots.net et al. 2021), there have been few recent attempts to analyze regional floristic patterns using the permanent plot data or to compare the region's dominant forest types.

Permanent plots are foundational to our understanding of tree diversity and composition across and within regions of the Amazon. For example, many studies using plot data have found that tree diversity generally increases with precipitation across the basin (Gentry 1982; Pitman et al. 2002; Esquivel‐Muelbert et al. 2017). Other studies conclude that different habitat types, namely floodplain forest and terra firme forest, are compositionally distinct and contribute to high beta and gamma diversity in the Amazon (Gentry 1992; Bredin et al. 2020; Householder et al. 2024). Whether or not these patterns hold within smaller geographic regions, for example in MDD, is not as well understood. Such analyses are possible and valuable in MDD where plots are numerous and there is a wide range of precipitation regimes and habitat types. However, potential bias in where plots are located within the region can skew our understanding of the region's forest dynamics.

Historically, most permanent forest dynamics plots have been installed in old‐growth or primary forests, or forests that were potentially subject to ancient human disturbance (McMichael et al. 2017). Thus, global plot networks (e.g., ForestPlots and ATDN) have largely ignored secondary and recently disturbed forest. Given that a large and growing proportion of the world's tropical forests are directly impacted by human activities (Lewis et al. 2015), this “majestic forest bias,” as Malhi et al. (2002) aptly described it, can misrepresent the true nature of today's tropical forests and lead to, for example, negative bias on growth estimates. To combat this, there has been a recent push to better represent secondary and disturbed forests within existing plot networks, and even to create entirely new networks dedicated to these forests. For example, the Tropical Managed Forests Observatory focuses on the effects of tree harvest on forest dynamics (Sist et al. 2015) and 2ndFOR focuses on the dynamics and ecosystem services of tropical secondary forests (Poorter et al. 2016). As global land‐use change and other anthropogenic impacts accelerate, it will be important to continue establishing plots within disturbed and managed forests to monitor these underrepresented forest types (Bongers et al. 2015). Although the southwestern Amazon, particularly MDD, contains a multitude of permanent forest dynamics plots, much of the region's forests are still poorly represented in plot databases, especially managed and disturbed forests.

Nearly 30% of the MDD region is terra firme forest dominated by Brazil nut trees ( *Bertholletia excelsa* , Lecythidaceae) (Chávez et al. 2012), and a large portion of these forests are actively managed for Brazil nut harvest. Although Brazil nut trees occur throughout much of the Amazon, MDD is one of the only regions where densities are high enough (1.3–1.5 adult trees per hectare; Rockwell et al. 2015) for commercial nut extraction from natural forest (Zuidema 2003), contributing to Peru's place as the second largest exporter of Brazil nuts behind only Brazil (Willem et al. 2019). As such, Brazil nuts are a vital economic resource for many families and communities within MDD. In fact, one study found that Brazil nuts comprise over half of the income for many households in the Brazil nut sector of MDD (Garrish et al. 2014), with another study highlighting a similar importance in northern Bolivia (Zuidema 2003). In MDD, most Brazil nut harvest occurs within managed concessions, which are areas of land granted by the government to an individual, community, or private entity (the “concessionaire”) over a long‐term contract that grants legal harvest rights to the concessionaire (Willem et al. 2019). Although there are some Brazil nut concessions within natural protected areas (e.g., in Tambopata National Reserve), most concessions are outside of protected areas and are subject to other anthropogenic forest disturbances (Willem et al. 2019). For example, Peru's national authority on forestry and wildlife, SERFOR, currently permits the harvest of up to 5 m^3^ ha^−1^ of timber (roughly 1–1.5 trees ha^−1^) within Brazil nut concessions, a quota that does not reset (Cossío‐Solano et al. 2011; Ramirez and Belcher 2020). Unfortunately, illegal logging is rampant in Peru and many concessionaires harvest beyond this quota, especially in more remote and/or poorly supervised concessions (Praeli 2019). Consequently, the majority of Brazil nut concessions have been subject to varying levels of human intervention beyond the harvest of Brazil nuts. Still, Brazil nut reliance has been shown to slow down deforestation since production is highest in intact forest (Willem et al. 2019), highlighting that Brazil nut concessions are not only economically important but helpful for forest conservation as well. Despite their ecological and economic importance, there have been few efforts to study the dynamics of Brazil nut forests in Peru, especially in concessions outside of protected areas that have been subject to additional human disturbances such as logging. As such, our current understanding of the region's forest dynamics may be skewed.

Selective logging is a major driver of forest degradation in the Amazon and is increasing in frequency (Asner et al. 2005; Lapola et al. 2023). Logging affects tropical forests not only through the direct harvest of trees but also through collateral damage to nontarget trees through felling and the construction of temporary roads used in timber removal (Uhl and Vieira 1989). Immediate effects of logging include a decrease in carbon stocks, an increase in tree mortality, and a decrease in tree recruitment (Asner et al. 2005; Mazzei et al. 2010; Roopsind et al. 2018), but these changes are typically offset within 15–20 years as long as logging intensity is low (≤ 4 trees ha^−1^). Tree diversity, on the other hand, has been found to show a strong resilience to selective logging. Indeed, multiple studies found no difference in tree alpha diversity between logged and unlogged forests in Borneo (Verburg and Van Eijk‐Bos 2003; Berry et al. 2008). How Amazon tree diversity responds to selective logging is not as well understood, in part due to the lack of continuously monitored forest dynamics plots in selectively logged forest.

Despite the prevalence of managed and disturbed forests in MDD, the vast majority of existing permanent plots in the region are within protected areas and are thus buffered from most direct human disturbance. These protected areas include Manu National Park, Tambopata National Reserve, the Los Amigos Conservation Concession, and the InkaTerra Ecotourism Concession. Although there are plots in Brazil nut forest in Tambopata National Reserve and harvesting occurs in nearby concessions, these plots are not subject to other anthropogenic activities such as logging. In summary, there is a lack of forest monitoring initiatives within managed and disturbed forests in MDD, thus limiting our knowledge of the region's forests as a whole.

The goal of this study is to characterize the floristics and forest dynamics of a disturbed plot in MDD and compare it to others in the region. We first describe a 1‐ha permanent forest dynamics plot located in a logged forest in the Brazil nut sector of MDD. The site was subject to low‐intensity selective logging around 25 years ago and is adjacent to deforested agricultural land and the edge of a large tract of contiguous forest dominated by Brazil nut concessions. We describe the plot's diversity, composition, structure, and short‐term (3 years) dynamics for the first time. By comparing the plot to other plots in the region, we also analyze regional‐scale patterns in diversity and composition, focusing primarily on precipitation effects on tree diversity and habitat type (i.e., floodplain vs. terra firme) on species composition. We hypothesized that plot alpha diversity would increase with precipitation and that there would be a clear difference in tree species composition between floodplain and terra firme plots. Finally, we evaluate if our new plot is qualitatively different from plots in unlogged, protected forests. Because logging at our site was low intensity and occurred > 20 years ago, we predicted that the diversity, composition, and short‐term dynamics of our new plot would all be comparable to other plots in the region.

## 2 Materials and Methods

### 2.1 Study Site

#### 2.1.1 Location

**FIGURE 1:**
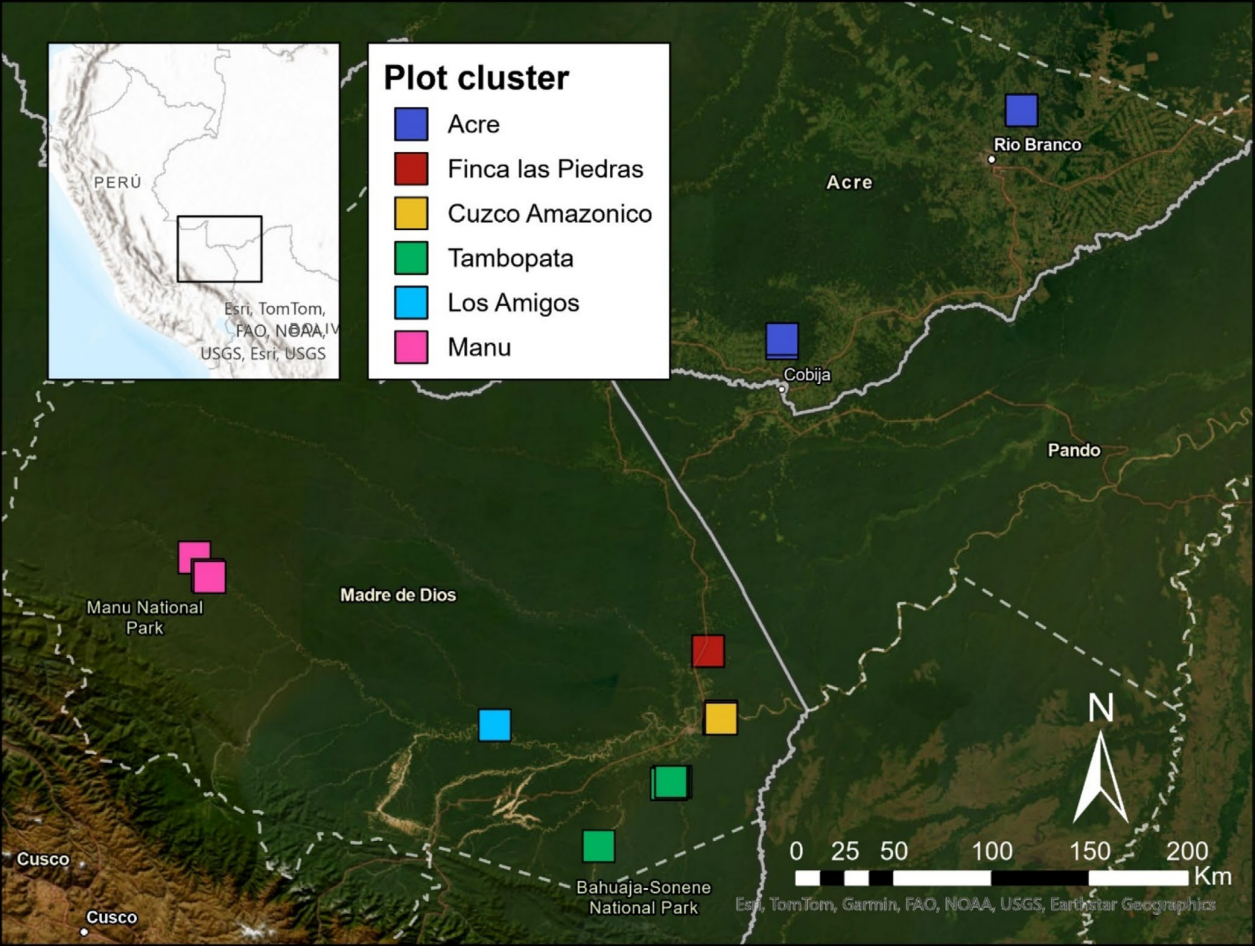
Map of the southwest Amazon basin indicating the location of the plots analyzed in this study. Squares and colors indicate plots and the geographic cluster in which the plots belong, respectively.

The new plot is located at Finca Las Piedras (FLP), a 54‐ha research station operated by the nonprofit organization Alliance for a Sustainable Amazon (ASA) in southeastern Peru (12°13′34.85″ S, 69°6′45.36″ W, Figure 1). FLP is privately owned, and the property is protected from most direct human disturbances. However, it is surrounded by privately owned land on its north, west, and south boundaries that are dominated by agriculture. On its eastern boundary, FLP borders Brazil nut concessions that continue east until the border with Bolivia, beyond which is the Reserva Nacional de Vida Silvestre Amazónica Manuripi, a protected area totaling 7470 km^2^ that allows communities to harvest Brazil nuts within its borders (Licona‐Vasquez et al. 2010). In summary, FLP is at the confluence of deforested agricultural land and millions of hectares of contiguous forest including > 1,000,000 ha of managed Brazil nut forest.

#### 2.1.2 Vegetation and Site History

FLP is on terra firme, is never inundated, and the majority of the property is typical Brazil nut forest with ~50 adult trees on the entire property (a density of ~0.92 adult trees per hectare). FLP also features a small “aguajal” swamp dominated by the aguaje palm ( *Mauritia flexuosa* , Arecaceae), abandoned pasture undergoing natural and assisted regeneration, and small‐scale agroforestry systems. Around 25 years ago, low‐intensity selective logging of high‐value timber species (e.g., *Cedrelinga cateniformis* [Fabaceae], *Dipteryx ferrea* [Fabaceae], *Cedrela odorata* [Meliaceae], and *Handroanthus serratifolius* [Bignoniaceae]) occurred at roughly one to two trees per hectare (G. Gallice, unpublished data). While we did not find any cut stumps within the plot, multiple cut stumps are within ~20 m of the plot boundary. Across the FLP property, there are multiple rutted tracks left from heavy machinery used to haul timber from the forest; however, several trees were felled but subsequently left in the forest due to having rotted trunks or otherwise low‐quality timber. Within the plot, there is at least one track ~120 m in length and ~4 m wide.

#### 2.1.3 Climate

In June 2017, a thermometer and manual rain gauge were installed at FLP and checked on a daily basis. Since then, mean annual precipitation (MAP) is 1980 mm, with > 85% of annual rainfall occurring between the months of October and April. Dry season months (May–September) receive a cumulative annual average of 286 mm precipitation. Minimum and maximum temperatures vary widely from month to month partially due to “friajes” southerly cold fronts, during the dry season/austral winter. Average maximum and minimum temperatures are less variable due to hotter temperatures in the dry season outside of friajes and are 27.74°C and 21.63°C, respectively (Figure S1).

### 2.2 Sampling

#### 2.2.1 Plot Installation

We installed the 1‐ha (100 × 100 m) plot (hereafter referred to as FLP‐01) at FLP in June 2021. The plot's location was chosen to avoid as many existing trails as possible and with each plot edge at least 100 m from a forest edge (or property boundary), to avoid as many edge (or future edge) effects as possible (Laurance et al. 2002). Once we decided the location of the plot's northwest corner, we demarcated 100‐m boundaries using a handheld compass to maintain a direct heading in each cardinal direction and, within the 1‐ha plot, marked 100 10 × 10 m subplots. We marked each 10‐m interval with a 1‐m PVC tube painted red and staked at least 20 cm into the ground.

#### 2.2.2 Stem Measurements

In the plot, we tagged, measured, mapped, and identified every self‐supporting woody stem ≥ 10 cm diameter at breast height (DBH) following standard protocols (Lopez‐Gonzalez et al. 2011). For trees with buttressed trunks or other irregularities, diameter was measured above or below the irregularities, and measurement height was noted. We collected voucher specimens for species identifications and stored vouchers in the natural history collection of ASA.

We also scored each tree for liana infestation, sun exposure, and canopy damage. Liana infestation scores were 0 for trees without lianas and 1, 2, 3, or 4 for trees with 1%–25%, 26%–50%, 51%–75%, or 76%–100% of their canopy covered by lianas, respectively. Sun exposure scores were from 1 to 5, with 1 for plants lacking direct sunlight except during sun flecks and 5 for canopy emergents. Crown damage scores were 0 for trees with intact crowns and 1, 2, 3, or 4 for trees with 1%–25%, 26%–50%, 51%–75%, or 76%–100% of the crown broken, respectively. Finally, we estimated the height of each tree.

In July 2024, we performed a complete recensus of the plot, remeasuring and rescoring each stem following the same protocols described above. We also recorded tree mortality by noting which trees had died since the previous census and their suspected cause of death. We also measured, tagged, mapped, identified, and scored each new recruit—that is, each stem that attained a DBH of ≥ 10 cm since the previous census. To summarize, plot censuses took place in the dry seasons of 2021 and 2024.

### 2.3 Acquiring Other Plot Data

We compared FLP‐01 to 21 other plots in the southwest Amazon. We identified 20 plots in the ForestPlots.net database (Lopez‐Gonzalez et al. 2011) from within MDD and neighboring Acre, Brazil, that have multiple censuses and where species lists are available for public use. Of these plots, 17 are from MDD and 3 are from Acre (Figure 1, Table 1). We also include data from an additional plot in the Tambopata National Reserve, TRC‐01. When searching for plots, we only chose plots from floodplain or terra firme forest and excluded plots in other habitats, such as one plot in Tambopata National Reserve in a swamp dominated by *Lueheopsis hoehnii* (Malvaceae), a species not found in any other plot in the region. All plots were installed and monitored following the same methods described above, including a minimum DBH threshold of 10 cm (Lopez‐Gonzalez et al. 2011). At each plot location, we extracted the estimated MAP from the WorldClim climate raster (Fick and Hijmans 2017) at 30 arc‐sec resolution.

**TABLE 1:** Metadata for each plot analyzed in the current study. Listed are the plot codes, plot names, principal investigators (PIs), latitude and longitude, cluster in which they belong, meters above sea level (m asl), habitat, plot size in ha, and the census year for each plot.

Plot code	Plot name	PIs	Lat	Lon	Cluster	m asl	Habitat	Size	Data year
ALM‐01	Altos de Maizal	J. Terborgh, N. Pitman	−11.8	−71.47	Manu	400	Terra firme	2	2008
CUZ‐01	Cuzco Amazonico 1	A. Monteagudo‐Mendoza, O. Phillips	−12.54	−69.06	Cuzco Amazonico	190	Floodplain	1	2008
CUZ‐02	Cuzco Amazonico 2	A. Monteagudo‐Mendoza, O. Phillips	−12.54	−69.06	Cuzco Amazonico	190	Floodplain	1	2008
CUZ‐03	Cuzco Amazonico 3	A. Monteagudo‐Mendoza, O. Phillips	−12.53	−69.05	Cuzco Amazonico	190	Floodplain	1	2008
CUZ‐04	Cuzco Amazonico 4	A. Monteagudo‐Mendoza, O. Phillips	−12.54	−69.05	Cuzco Amazonico	190	Floodplain	1	2008
FLP‐01	Finca las Piedras	G. Gallice, R. Fortier	−12.23	−69.11	Finca las Piedras	250	Terra firme	1	2024
LAS‐02	Jacaratia Los Amigos	F. Cornejo Valverde, N. Pitman, O. Phillips	−12.57	−70.09	Los Amigos	235	Floodplain	1	2008
MNU‐05	Manu, alluvial Cocha Cashu Trail 12	J. Terborgh, O. Phillips, R. Brienen	−11.88	−71.41	Manu	347	Floodplain	2.25	2008
MNU‐06	Manu, alluvial Cocha Cashu Trail 2 and 31	F. Cornejo Valverde, J. Terborgh, O. Phillips, R. Brienen	−11.89	−71.40	Manu	345	Floodplain	2.25	2008
POR‐01	RESEX Chico Mendes: Seringal Porongaba 1	M. Silveira	−10.82	−68.77	Acre	268	Terra firme	1	2009
POR‐02	RESEX Chico Mendes: Seringal Porongaba 2	M. Silveira	−10.8	−68.77	Acre	268	Terra firme	1	2009
RFH‐01	Reserva Florestal Humaita	F. Brown, M. Silveira	−9.75	−67.67	Acre	176	Terra firme	1	2011
TAM‐01	Tambopata plot zero	A. Monteagudo‐Mendoza, O. Phillips, R. Vasquez Martinez, T. Baker, T. Feldpausch	−12.84	−69.29	Tambopata	205	Terra firme	1	2008
TAM‐02	Tambopata plot one	A. Monteagudo‐Mendoza, O. Phillips, R. Vasquez Martinez, T. Feldpausch	−12.83	−69.29	Tambopata	210	Terra firme	1	2008
TAM‐04	Tambopata plot two swamp edge clay	A. Monteagudo‐Mendoza, O. Phillips, R. Vasquez Martinez, T. Feldpausch	−12.84	−69.28	Tambopata	210	Terra firme	0.42	2008
TAM‐05	Tambopata plot three	A. Monteagudo‐Mendoza, O. Phillips, R. Vasquez Martinez	−12.83	−69.27	Tambopata	220	Terra firme	1	2008
TAM‐06	Tambopata plot four	A. Monteagudo‐Mendoza, O. Phillips, R. Vasquez Martinez, T. Erwin	−12.84	−69.30	Tambopata	200	Floodplain	1	2008
TAM‐07	Tambopata plot six	A. Monteagudo‐Mendoza, O. Phillips	−12.83	−69.26	Tambopata	225	Terra firme	1	2011
TAM‐08	Tambopata plot seven	A. Monteagudo‐Mendoza, O. Phillips	−12.83	−69.27	Tambopata	220	Terra firme	1	2011
TAM‐09	Tambopata plot eight	J. Silva Espejo, O. Phillips, Y. Malhi	−12.83	−69.28	Tambopata	199	Terra firme	1	2010
TRC‐01	Tambopata Research Center	V. Swamy	−13.12	−69.62	Tambopata	240	Floodplain	1	2020

### 2.4 Data Analysis

#### 2.4.1 Diversity and Composition

We first tabulated the number of stems and the species richness for each 1‐ha plot. Some plots, however, are smaller or larger than 1 ha, limiting our ability to directly compare species richness between all plots. We therefore controlled for the number of stems per unit area by also calculating the Shannon diversity index and species evenness for each plot. We then analyzed if there is a correlation between MAP and plot diversity using Spearman's rank correlation. To visualize how species richness increases with the number of stems, we generated rarefaction curves for each plot. In generating rarefaction curves, we used a step size of five stems and tabulated species richness at each step until the maximum number of stems in each plot was reached.

To explore compositional differences of plots within the region, we performed an ordination using nonmetric multidimensional scaling (NMDS) based on Bray–Curtis community dissimilarities of species abundances. To determine an optimal number of NMDS dimensions, we ran 1000 trials using 1–10 dimensions to yield different stress values. We chose three dimensions, which was the lowest number of dimensions that yielded a stress value ≤ 0.1 (Figures S2–S4). We then performed a cluster analysis based on the Bray–Curtis dissimilarities to better visualize compositional groupings of all plots. To determine if compositional differences are correlated with geographic distances and precipitation differences between plots, we performed simple Mantel tests. We used the R package “vegan” (Oksanen et al. 2025) for diversity and compositional analyses.

We then sought to characterize which tree species are associated with floodplain and terra firme forests with an indicator species analysis. To do so, we performed a multilevel pattern analysis with the R package “indicspecies” (de Cáceres and Legendre 2009). Finally, to further explore which tree species are important in each habitat type, we pooled data for floodplain and terra firme plots and calculated importance value indices (IVI) for each species in both habitats. IVI were calculated as (relative density + relative basal area)/2.

#### 2.4.2 Structure and Dynamics

We calculated stem density for each plot as the number of stems per hectare. To characterize differences in DBH distributions between plots, we generated violin plots and calculated the mean, maximum, and interquartile range of tree diameters. We then calculated aboveground biomass (AGB) using measured tree diameters, estimated tree heights, and estimated wood densities. Tree heights for each individual in all plots other than FLP‐01 were estimated using a general allometric model from the R package BIOMASS (Chave et al. 2014; Réjou‐Méchain et al. 2017) that calculates tree heights based on DBH. Wood densities for each species were taken from a published wood density database (Zanne et al. 2009). When a species‐level wood density was not available, we used the genus‐ or family‐level wood density average instead. Finally, we estimated AGB for each tree using an allometric model from the R package BIOMASS (Chave et al. 2014, Réjou‐Méchain et al. 2017). For FLP‐01, we calculated the change in total AGB between censuses and compared this change to that of other plots in MDD for which data is published.

We characterized stem turnover within the FLP‐01 plot by calculating the annual mortality (*λ*) and recruitment (*μ*) rates using the following equations adapted from Phillips and Gentry (1994) and Lewis et al. (2004):

$$\lambda =\left[\ln\left(N_{0}\right)-\ln\left(N_{s}\right)\right]/t$$

$$\mu =\left[\ln\left(N_{s}+N_{r}\right)-\ln\left(N_{s}\right)\right]/t$$

where *N*_0_ is the number of stems in the original census, *N*_s_ is the number of original stems surviving to the final census, *N*_r_ is the number of new recruits in the final census, and *t* is the number of years between censuses. We then took the average of *λ* and *μ* to calculate total stem turnover between the first and second censuses. We compared stem turnover of FLP‐01 to that of other plots in MDD for which data are published.

All analyses were performed using R statistical software (R Core Team 2021).

## 3 Results

### 3.1 FLP Plot Characteristics

FLP‐01 censuses took place in 2021 and 2024, where we measured, tagged, mapped, and identified 549 and 582 trees ≥ 10 cm DBH in the first and second censuses, respectively. There were 173 and 181 species in the first and second censuses, respectively, in 48 families. The three most speciose families were Moraceae (20 spp.), Fabaceae (18 spp.), and Lauraceae (18 spp.). The five most common species were *Siparuna decipiens* (Siparunaceae, 29 individuals in the second census), *Euterpe precatoria* (Arecaceae, 28 individuals), *Galipea trifoliata* (Rutaceae, 23 individuals), *Pausandra trianae* (Euphorbiaceae, 20 individuals), and *Bixa excelsa* (Bixaceae, 17 individuals), which together comprised just over 20% of all individuals in the plot.

In the second census of FLP‐01, tree diameters ranged from 10 to 196.3 cm, with a mean diameter of 21.4 cm (Figure S5). The four species with the largest total basal area in the second census were *Bertholletia excelsa* (Lecythidaceae, 40,612 cm^2^, 12.3% of total basal area), *Protium altissimum* (Burseraceae, 21,495 cm^2^, 6.5%), *Ficus gomelleira* (Moraceae, 16,241 cm^2^, 4.9%), and *Iryanthera laevis* (Myristicaceae, 14,329 cm^2^, 4.3%) (Table S1). We estimated AGB to be 285 and 316 t for the first and second censuses, respectively, translating to a total increase of 31 t of AGB or an annual increase of 10.3 t. Between the first and second censuses of FLP‐01, a total of 22 individuals died and 55 new individuals recruited into the measurable cohort. This translates to an annual stem turnover of 2.23% year^−1^, with annual rates of mortality and recruitment of 1.36% year^−1^ and 3.31% year^−1^, respectively.

### 3.2 Regional Plot Characteristics

**FIGURE 2:**
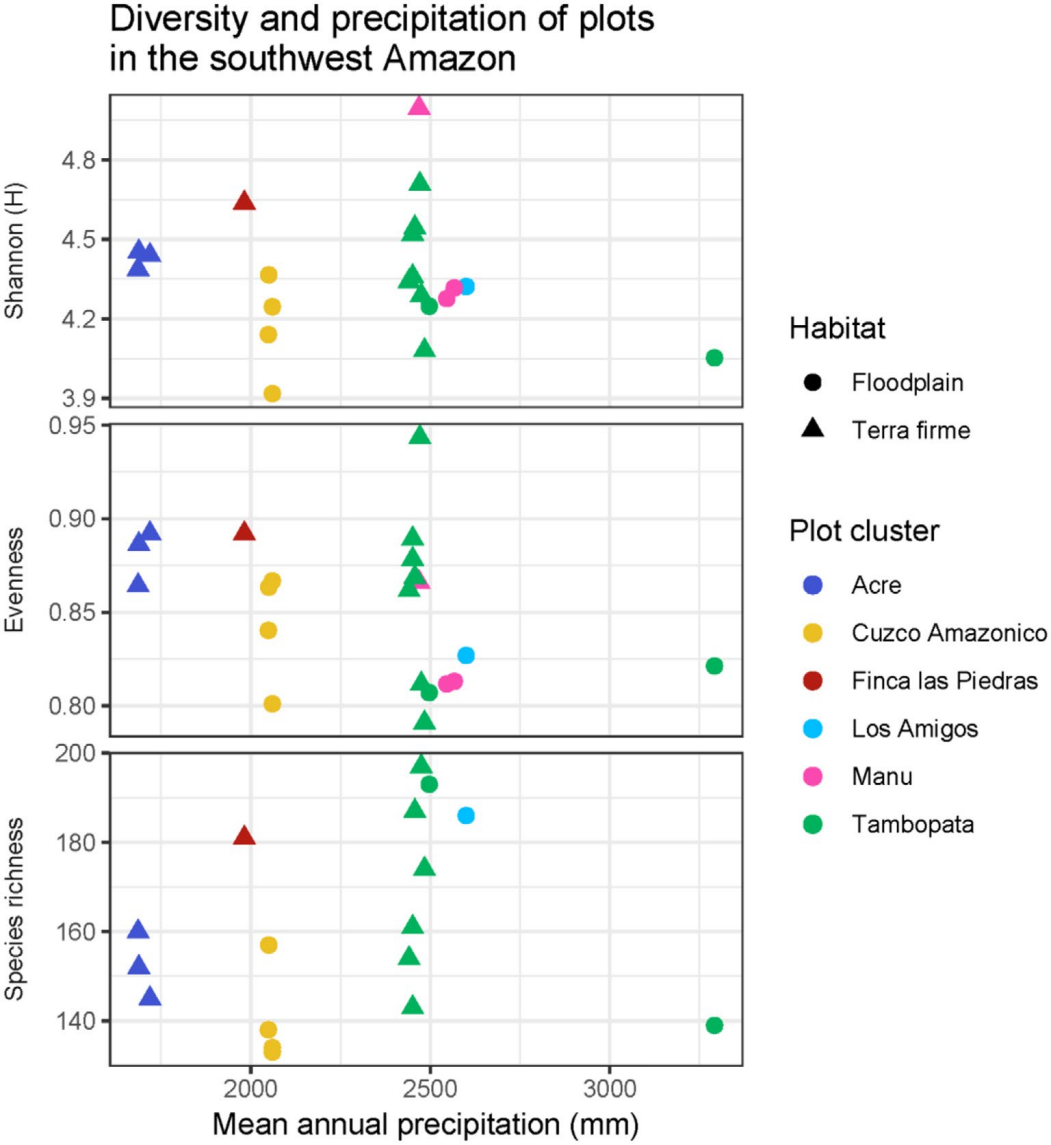
Scatterplot of three diversity metrics as a function of mean annual precipitation (MAP) for permanent forest dynamics plots in the southwest Amazon. Diversity indices are Shannon index (*H*), species evenness, and species richness. Colors represent the plot clusters as shown in Figure 1. Circles represent floodplain plots, whereas triangles represent terra firme plots. Spearman's rank correlation found no significant relationship between MAP and both Shannon index and evenness (*p* values ≥ 0.20 for both correlations). Similarly, there was no significant correlation between MAP and species richness for 1‐ha plots (*p* value = 0.12). Note that for species richness, four plots that are greater than or less than 1‐ha are not shown.

Among all 1‐ha plots, FLP‐01 is the fifth most speciose plot (Figure 2, Table 2). However, when controlling for the number of stems in each plot using the Shannon diversity index, FLP‐01 is the third most diverse plot. Similarly, species evenness of FLP‐01 is the third highest among all plots. Spearman's rank correlation revealed no significant correlation between MAP and Shannon index (Spearman's rho = −0.28, *p* value = 0.22) or species evenness (Spearman's rho = −0.29, *p* value = 0.20) among plots. Similarly, when analyzing only 1‐ha plots (17 of 21 plots), there was no correlation between MAP and species richness (Spearman's rho = 0.39, *p* value = 0.12). Rarefaction curves revealed slight differences among all plots in their species accumulation curves (Figure S6). One plot in Manu and one in Tambopata yielded the most rapid increases in species with an increased number of stems. Meanwhile, the plots at Cuzco Amazonico had, on average, the slowest increases in species. Unsurprisingly, the rarefaction curves suggest that increased sampling would yield additional species (i.e., species accumulation curves did not reach asymptotes).

**TABLE 2:** Plot statistics. Listed are the plot codes, number of species (Spp), Shannon's index (*H*′), number of stems, species evenness, mean annual precipitation (MAP) in mm, and aboveground biomass (AGB) in metric tons. Additional data for Finca las Piedras and Tambopata plots include annual AGB change in metric tons ha^−1^, annual rate of mortality (*λ*), annual rate of recruitment (*μ*), and annual stem turnover.

Plot	Spp	*H*′	Stems	Evenness	MAP	AGB	Annual AGB change	*λ*	*μ*	Stem turnover
FLP‐01	181	4.64	583	0.892	1982	317	10.3	1.36	3.31	2.23
TAM‐01*	174	4.08	620	0.791	2484	269	2.5	1.91	1.83	1.87
TAM‐02*	197	4.29	682	0.812	2475	288	−3.4	2.42	2.47	2.45
TAM‐04*	147	4.71	303	0.943	2471	333	1.3	2.12	1.40	1.76
TAM‐05*	161	4.52	535	0.889	2451	287	3.5	1.99	1.84	1.91
TAM‐06*	193	4.25	663	0.807	2497	340	5.4	1.56	2.13	1.85
TAM‐07*	154	4.34	509	0.862	2441	267	−6.6	3.10	2.66	2.88
TAM‐08*	143	4.36	517	0.878	2451	246	0.8	2.57	2.21	2.39
TAM‐09*	187	4.54	556	0.869	2457	271	−9.6	2.00	1.28	1.64
TRC‐01	139	4.05	535	0.821	3292	267	−4.6	2.37	2.16	2.25
ALM‐01	320	5.00	1324	0.866	2469	296				
CUZ‐01	134	4.25	435	0.867	2060	269				
CUZ‐02	133	3.92	556	0.801	2060	291				
CUZ‐03	138	4.14	505	0.840	2049	280				
CUZ‐04	157	4.37	602	0.863	2050	306				
LAS‐02	186	4.32	592	0.827	2600	284				
MNU‐05	194	4.28	1234	0.812	2546	342				
MNU‐06	202	4.32	1212	0.813	2567	322				
POR‐01	152	4.45	551	0.886	1688	367				
POR‐02	160	4.39	520	0.864	1686	249				
RFH‐01	145	4.44	383	0.892	1719	304				

*Note:* Asterisks next to plot codes indicate that the additional data for those plots were gathered from Pallqui et al. (2014).

**FIGURE 3:**
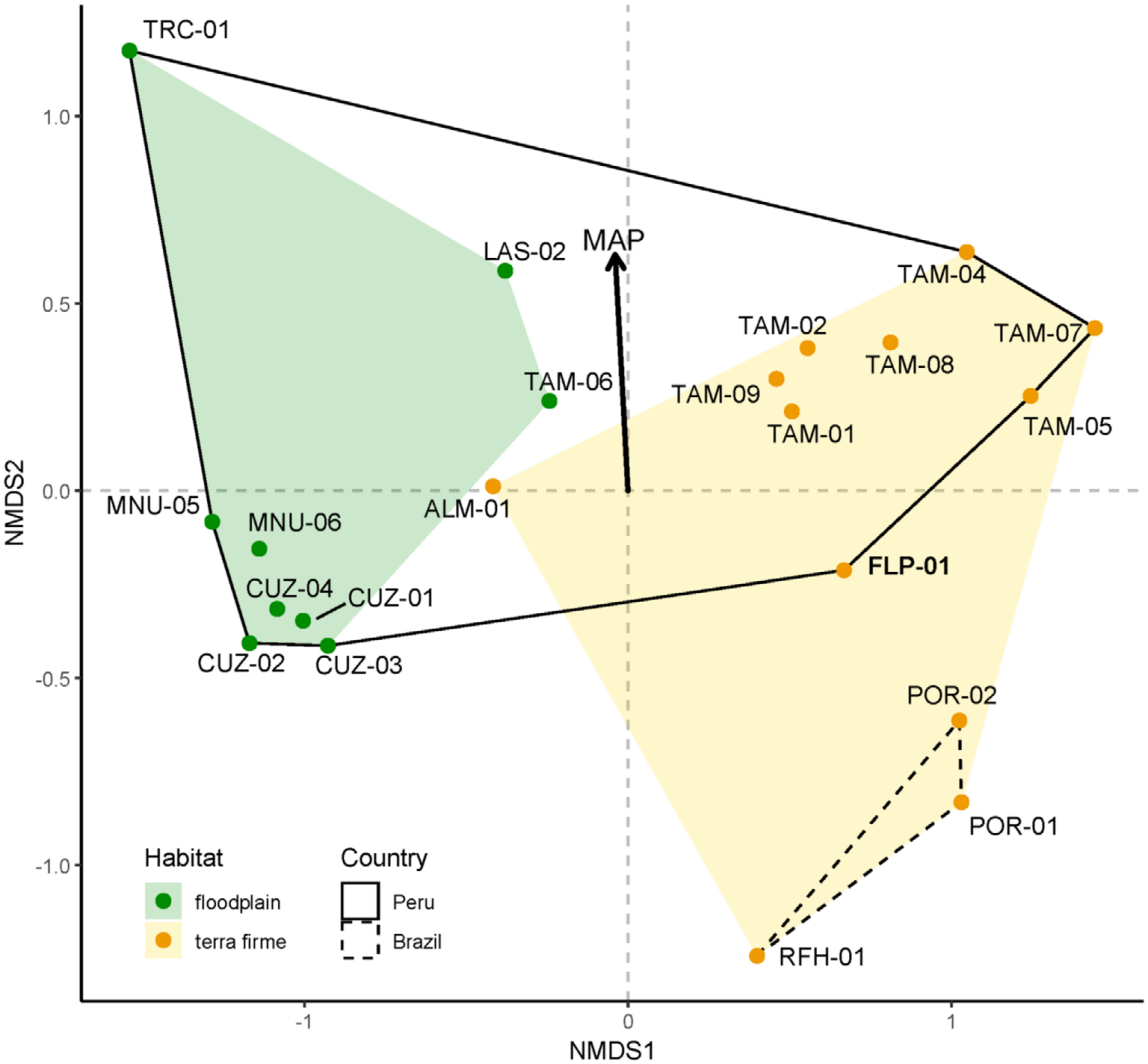
Nonmetric multidimensional scaling (NMDS) ordination for permanent forest inventory plots in the current study (stress value = 0.07). Species abundances were used to compute the ordination in three dimensions. Green and yellow dots (and polygons) indicate floodplain and terra firme plots. The solid‐line polygon circumscribes plots in Peru, whereas the dotted‐line polygon circumscribes plots in Brazil. The arrow indicates the direction in ordination space in which mean annual precipitation (MAP) changes most rapidly, whereas the length of the arrow indicates the rate of change. FLP‐01 is shown in bold text.

The NMDS ordination showed a clear separation in species composition between plots in floodplain versus terra firme forests along the first ordination axis (Figure 3, Figure S4). The ordination and cluster dendrogram (Figure S7) both showed that FLP‐01 is more similar to two plots in Acre, Brazil, than it is to most of the plots in MDD. The cluster dendrogram also showed that compositional clusters were mostly correlated with geographic clusters; however, the Tambopata plots were separated into two distinct compositional groups. Simple Mantel tests revealed that compositional differences between plots are not significantly correlated with geographic distances (Mantel statistic *r* = 0.12, *p* value = 0.14). They are, however, marginally significantly correlated with precipitation differences (Mantel statistic *r* = 0.14, *p* value = 0.07).

In total, there were 22 species that were significantly associated with floodplain plots and 17 that were significantly associated with terra firme plots (Table S2). The three most significant floodplain species were *Brosimum alicastrum* (Moraceae), *Astrocaryum murumuru* (Arecaceae), and *Poulsenia armata* (Moraceae). Meanwhile, the three most significant terra firme species were *Oenocarpus bataua* (Arecaceae), *Glycydendron amazonicum* (Euphorbiaceae), and *Pseudolmedia macrophylla* (Moraceae). Note that two taxa had higher significance scores for terra firme plots, *Inga* “indet” (Fabaceae) and *Pouteria* “indet” (Sapotaceae). These undoubtedly represent multiple species and thus do not indicate an association of any one species of *Inga* or *Pouteria* to terra firme forest. The three species with the highest IVI for pooled floodplain plot data were *Iriartea deltoidea* (Arecaceae), *Pseudolmedia laevis* (Moraceae), and *Otoba parvifolia* (Myristicaceae) (Table S3). The three species with the highest IVI for pooled terra firme plot data were *Iriartea deltoidea* (Arecaceae), *Pourouma minor* (Urticaceae), and *Protium altissimum* (Burseraceae) (Table S3).

**FIGURE 4:**
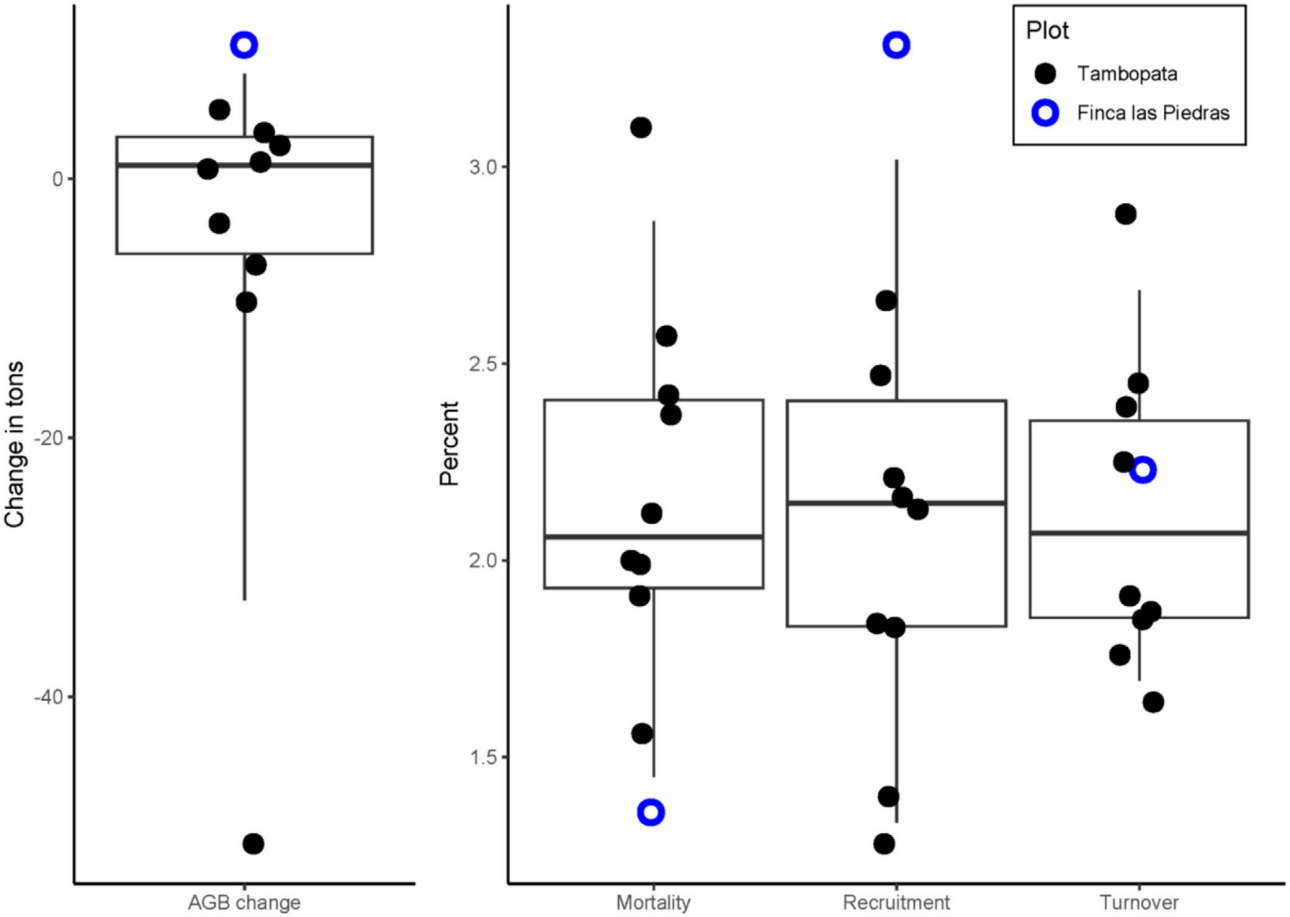
Boxplots showing aboveground biomass (AGB) change and annual rates of mortality, recruitment, and turnover of Tambopata and Finca las Piedras plots. Black circles are Tambopata plots and open blue circles are the Finca las Piedras plot. For each variable, horizontal lines indicate the mean, boxes indicate the interquartile ranges, and whiskers indicate the 95% confidence intervals.

Stem density within plots ranged from 383 to 721 stems per hectare (Figure S8). Meanwhile, maximum tree DBH within plots ranged from 99.4 to 197.2 cm, the highest DBH being a *Ficus schultesii* (Moraceae) in plot TAM‐06 (Figure S9). AGB in FLP‐01 was similar to that of other plots in the MDD and Acre regions (Figure S10). However, the AGB increase of 10.3 t year^−1^ in FLP‐01 is higher than that of other terra firme plots. Indeed, among nine plots in the Tambopata National Reserve, Pallqui et al. (2014) reported an average annual AGB change of −0.08 t year^−1^ between the years 2003–2011, with a minimum and maximum of −9.6 and 5.4 t year^−1^, respectively (Table 2, Figure 4). Similarly, the annual AGB change of TRC‐01 was −4.6 t year^−1^. Meanwhile, the mortality rate of FLP‐01 was low while the recruitment rate was high, but the overall stem turnover was near average (Pallqui et al. 2014; Table 2, Figure 4).

## 4 Discussion

In this paper we described a new 1‐ha permanent forest dynamics plot, FLP‐01, for the first time and compared its diversity, structure, and composition with other plots in the southwest Amazon. Despite its history of logging and its close proximity to deforested agricultural land, we found many similarities between FLP‐01 and plots in old‐growth, protected forests. Tree diversity in FLP‐01 was relatively high compared to other plots in the region; indeed, FLP‐01 was the third most diverse plot out of 21 based on the Shannon diversity index (Table 2, Figure 2). This is in accordance with previous studies that investigated the effect of selective logging on tropical tree diversity. For example, two studies found that the diversity of trees > 10 cm DBH in selectively logged forests in Borneo was no different than in nearby protected forests (Verburg and Van Eijk‐Bos 2003; Berry et al. 2008). In fact, Berry et al. (2008) found that the diversity of small trees (2.5–10 cm DBH) at a landscape scale was higher in the logged forest than in the protected forest. Similar studies are severely lacking in the Amazon (DeArmond et al. 2023). It is possible that the diversity of small trees in logged plots such as FLP‐01 is higher than that of unlogged plots in the region, due to an increased number of small stems resulting from high recruitment in logging gaps. However, most permanent plots do not inventory trees < 10 cm DBH, limiting our ability to perform such analyses. The exclusion of small trees in most plots is due to the expensive and time‐consuming nature of sampling smaller tree size classes. However, small tree diversity is often an important indicator of forest health, especially in disturbed forests (Rosenfield et al. 2023). Therefore, it will be important to consider including small trees and other understory vegetation when installing new plots or when comparing disturbed and old‐growth forests. Instead of sampling smaller stems throughout entire plots, stratified approaches (i.e., in smaller subplots) could reduce effort while still capturing important understory dynamics.

Our analyses found no significant relationship between precipitation and 1‐ha plot species richness (Figure 2). Similarly, there was no significant relationship between precipitation and Shannon diversity or species evenness (Figure 2). This is contrary to many studies that have found strong relationships between alpha diversity and precipitation over broader geographic scales. Indeed, tree diversity in the Neotropics as a whole generally increases with precipitation (Gentry 1982; Pitman et al. 2002; Esquivel‐Muelbert et al. 2017). Our contrasting results are likely due to analyzing fewer plots, a smaller geographic region, and/or a narrower range of MAP among plots. The range of MAP in other studies is ~1000 to > 4000 mm, whereas in our study the range of MAP was 1686–3300 mm. Given these constraints, it is perhaps unsurprising that there was no precipitation effect on alpha diversity of our small pool of plots across a smaller geographical region.

Compositionally, FLP‐01 is more similar to two plots in Brazil than it is to most of the MDD plots (Figure 3, Figure S7). This similarity is despite the fact that FLP‐01 is more than twice the distance to the nearest Brazilian plots (160 km) than to the nearest terra firme plot in MDD (70 km) (Figure 1). This was supported by a simple Mantel test, which showed that geographic distance does not correlate with compositional difference between plots—that is, just because two plots are far from each other does not mean they are inherently different compositionally. Notably, MAP at FLP‐01 is more similar to that of the Brazilian plots than to that of any terra firme plots in MDD (Table 2, Figure 2). This was also supported by a simple Mantel test which showed a marginally significant correlation between the difference in MAP and species composition between plots. FLP‐01 could therefore represent an important transitional forest from the wetter plots in MDD near the base of the Andes to the drier plots of Acre, Brazil. As an example, one abundant species in FLP‐01, *Galipea trifoliata* (Rutaceae), is absent in all other plots except for two plots in Acre where it is also common. The same is true for species common in the surrounding forest at FLP, such as *Geissospermum reticulatum* (Apocynaceae) and *Esenbeckia almawillia* (Rutaceae) (R. Fortier, personal observation), which are uncommon at the other Peruvian plot localities but common in neighboring Brazil and Bolivia.

We estimated that the AGB of FLP‐01 increased by 10.3 t year^−1^ over 3 years. This rate of AGB increase is nearly twice that of the highest reported increase among plots in Tambopata from 2003 to 2011 (Pallqui et al. 2014; Table 2). It is important to note that our findings are from a short period of just 3 years. It is likely that, given more time, the estimated AGB change of 10.3 t year^−1^ in FLP‐01 will decrease as larger trees naturally die and more census data is gathered. In addition, FLP‐01 census years were from 2021 to 2024, over a decade later than the Tambopata plot censuses (although the TRC‐01 censuses were from 2009 to 2020). There were major droughts in 2005 and 2010 (Chavez Michaelsen et al. 2020), which could have impacted carbon stocks during the Tambopata census years. Indeed, another study found that the 2015 drought in the Amazon led to a decrease in carbon accumulation across the Amazon basin, and this decrease was more prominent in regions with a strong dry season such as MDD (Bennett et al. 2023). Therefore, AGB change in the Tambopata plots was likely influenced by one or two major droughts between census years.

The annual rates of mortality and recruitment at FLP‐01 were 1.36% year^−1^ and 3.31% year^−1^, respectively. Comparing these to plots in Tambopata reveals a relatively low mortality rate and a relatively high recruitment rate at FLP‐01 (Pallqui et al. 2014; Table 2). Logged forests have been found to have exceptionally high rates of recruitment following logging events; indeed, logging gaps often mimic natural treefall gaps, with the resulting increase in solar irradiance leading to increased growth rates of trees (Herault et al. 2010) and helping to maintain the diversity of pioneer tree species and lianas (Schnitzer and Carson 2001). However, the initial boost in recruitment decreases through time, reaching similar levels to unlogged forest after 15 years (de Avila et al. 2015). Therefore, the high recruitment at FLP‐01 is likely not due to logging since trees were felled there ~25 years ago. Instead, it is more likely due to the short census interval compared to the Tambopata plots. In addition, similar to AGB change, mortality and recruitment rates in the Tambopata plots were likely higher and lower, respectively, than in FLP‐01 due to major drought events between census years (Bennett et al. 2023).

Although our study and others found no significant effect of logging on tree diversity or composition, it is important to note that logging, even at low intensities, can lead to the “commercial extinction” of certain species—that is, a species whose population is so depleted that it is no longer viable to harvest. One such species, mahogany ( *Swietenia macrophylla* , Meliaceae), has historically been one of the most valuable Amazonian timber species, but harvestable timber stocks are now virtually absent in Peru and Bolivia (Kometter et al. 2004). Mahogany, along with other desirable timber species, is therefore considered endangered due to logging activities throughout the Amazon. At high intensities, logging can lead to changes in species composition (de Avila et al. 2015) and degraded timber stocks (Roopsind et al. 2018) that can persist for multiple centuries.

One goal of our study was to bolster the representation of managed forests in plot networks due to the prevalence of managed Brazil nut concessions in the southwestern Amazon. At FLP, Brazil nut harvest occurs each year, including from two trees within FLP‐01. Brazil nut harvest also occurs in surrounding concessions. Of the other 20 plots analyzed in this study, nine contain Brazil nut trees and are likely influenced by annual Brazil nut harvests. For example, although Brazil nut harvest is not done within the property of the Tambopata plots, concessionaires harvest Brazil nuts in surrounding concessions. Because most Brazil nut forests in MDD are under active management by concessionaires (Willem et al. 2019), these plots are important to understand how Brazil nut tree dynamics, such as recruitment, are influenced by commercial harvesting. Indeed, one study found that juvenile Brazil nut trees were rare or absent in managed Brazil nut forests, indicating low to no recruitment when Brazil nut harvest is persistent (Peres et al. 2003). Similarly, another study found relatively low densities of juvenile Brazil nut trees in logged forests (Rockwell et al. 2017) compared to densities of juveniles found by another study in unlogged forests (Scoles and Gribel 2011). Although these studies may suggest a potential future decline of adult Brazil nut trees as logging continues, a scarcity of juveniles is common in some Brazil nut forests, even those not subject to current human disturbance (Scoles and Gribel 2011). Ultimately, decoupling Brazil nut tree demography from human disturbance is challenging due to the long occupation of the Amazon by humans. In fact, humans have likely been influencing Brazil nut populations for millennia (Mori and Prance 1990; Thomas et al. 2015; Levis et al. 2017). Nevertheless, by bolstering the representation of Brazil nut forests in public plot databases, the new FLP‐01 plot will help with the continued study of Brazil nut forest dynamics.

Not only is FLP‐01 valuable for future studies concerned with regional forest dynamics, but it also provides an important foundation for studies at its host site. Indeed, many long‐term monitoring projects at FLP will benefit from FLP‐01, for instance the Lepidoptera Diversity and Biology Project that is managed by the ASA and based at FLP. One of the key goals of this project is to document Lepidoptera–host plant interactions (Nakahara et al. 2022; Corahua‐Espinoza et al. 2022) and, to date, > 200 new Lepidoptera host plant records have been documented at FLP (G. Gallice, unpublished data). FLP‐01 provides an intriguing opportunity to expand the documentation of host plant records to large trees and even other insect orders. FLP's established infrastructure, including FLP‐01, a newly constructed laboratory equipped for DNA sequencing using nanopore technology, and the expertise of its Lepidoptera team demonstrate its potential as a world‐class plant–insect interaction study site.

FLP‐01 provides a diverse array of advantages for studies at both the regional and local scales. It represents an important transition forest between plots elsewhere in MDD and plots in southeastern Brazil, filling an important geographical gap in the distribution of permanent forest dynamics plots. It also bolsters the representation of disturbed and managed forests within long‐term monitoring initiatives. Indeed, data from FLP‐01 is currently hosted on the ForestPlots.net database, so it can be readily incorporated into future large‐scale studies of tropical forest dynamics. Continued monitoring of disturbed and managed forests will be important to further enhance our knowledge of the Amazon's forests. Finally, FLP‐01 can be used as a resource by researchers in studies on plant‐insect interactions, among other things. In summary, FLP‐01 is an important addition to the publicly available forest dynamics plots data and will help to bolster research in Peru's “capital of biodiversity.”

## Author Contributions

**Riley P. Fortier:** conceptualization (equal), data curation (lead), formal analysis (lead), funding acquisition (equal), investigation (lead), methodology (lead), project administration (lead), resources (equal), supervision (equal), validation (equal), visualization (lead), writing – original draft (lead), writing – review and editing (equal). **Thalia Corahua‐Espinoza:** data curation (equal), formal analysis (equal), investigation (equal), methodology (equal), writing – review and editing (equal). **Varun Swamy:** investigation (equal), methodology (equal), resources (supporting). **Kenneth J. Feeley:** conceptualization (equal), project administration (equal), resources (equal), supervision (equal), validation (equal), writing – review and editing (equal). **Geoffrey R. Gallice:** conceptualization (equal), funding acquisition (equal), project administration (equal), resources (lead), supervision (equal), writing – review and editing (equal).

## Conflicts of Interest

The authors declare no conflicts of interest.

## Supporting information


Appendix S1

## Data Availability

Climate data can be gathered from WorldClim (Fick and Hijmans 2017). Code used for statistical analyses and data for plots FLP‐01 and TRC‐01 are available from a GitHub repository: https://github.com/rpfortier/MDD_plot_comparisons. Restrictions apply to the availability of data for all other plots, which are available for registered users at ForestPlots.net with attribution to the data owners.
