# Supplementary material for: Insights From a New 1‐ha Permanent Forest Plot Reveal Differences Between Habitat Type and Similarities Between Forest Type in the Southwestern Amazon
Source: Ecol Evol. 2025 May 22;15(5):e71476. doi: 10.1002/ece3.71476 (PMC12098055; doi:10.1002/ece3.71476)
Supplement: Supplementary file 1 — Appendix S1 [file ECE3-15-e71476-s001.docx]

Supplementary Materials for:

**Insights from a new 1-ha permanent forest plot reveal differences between habitat type and similarities between forest type in the southwestern Amazon**


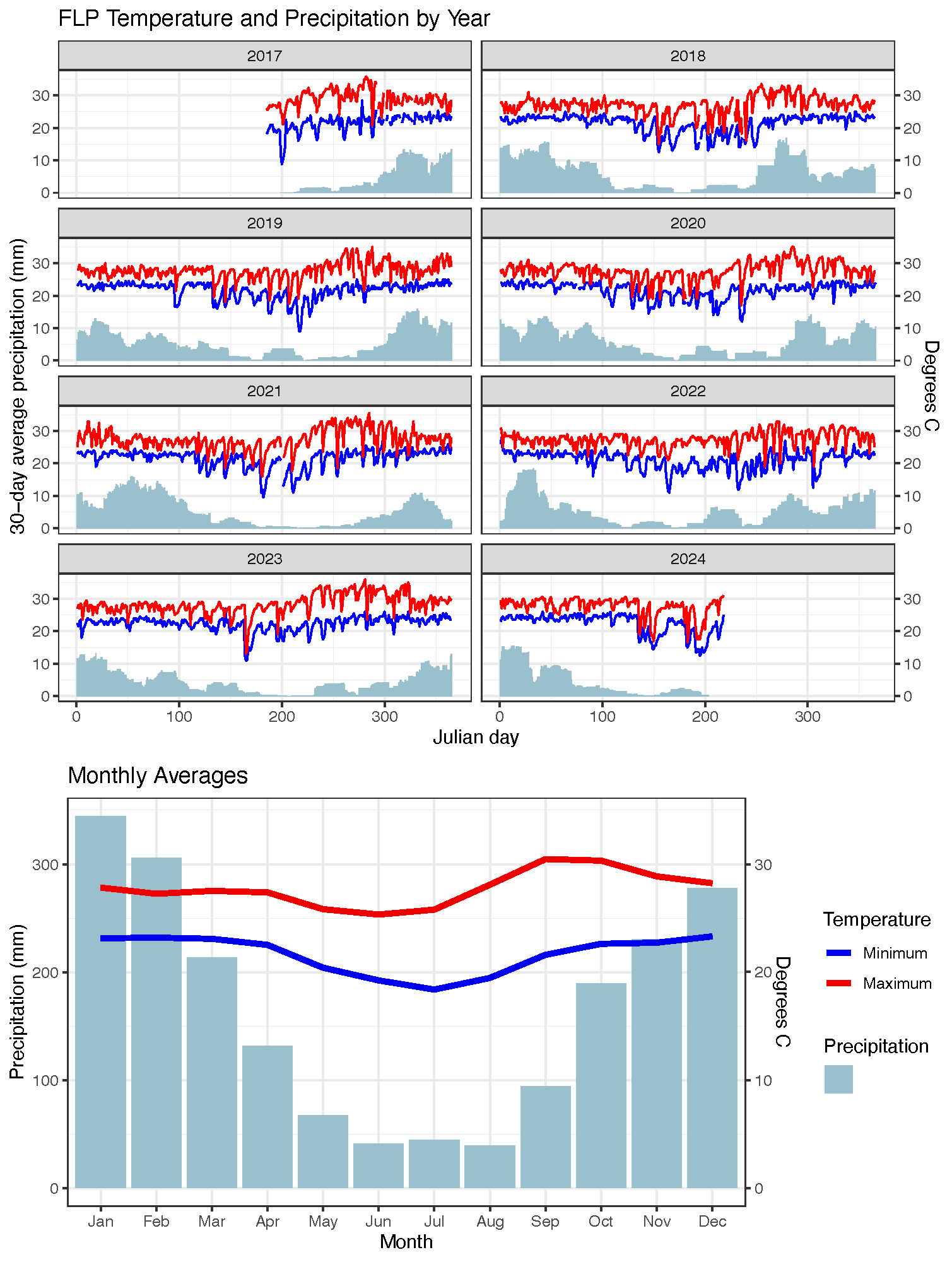


**Figure S1**: Daily and annual temperature and precipitation data from Finca las Piedras, MDD, Peru. Precipitation (light blue bars) is shown in millimeters as indicated by the Y-axes on the left. Maximum (red) and minimum (blue) temperatures are shown in degrees Celsius as indicated by the Y-axes on the right. The top panels show the 30-day rolling average precipitation and daily temperature values for each year starting on 4 July 2017, while the bottom panel shows monthly averages across all years.

**
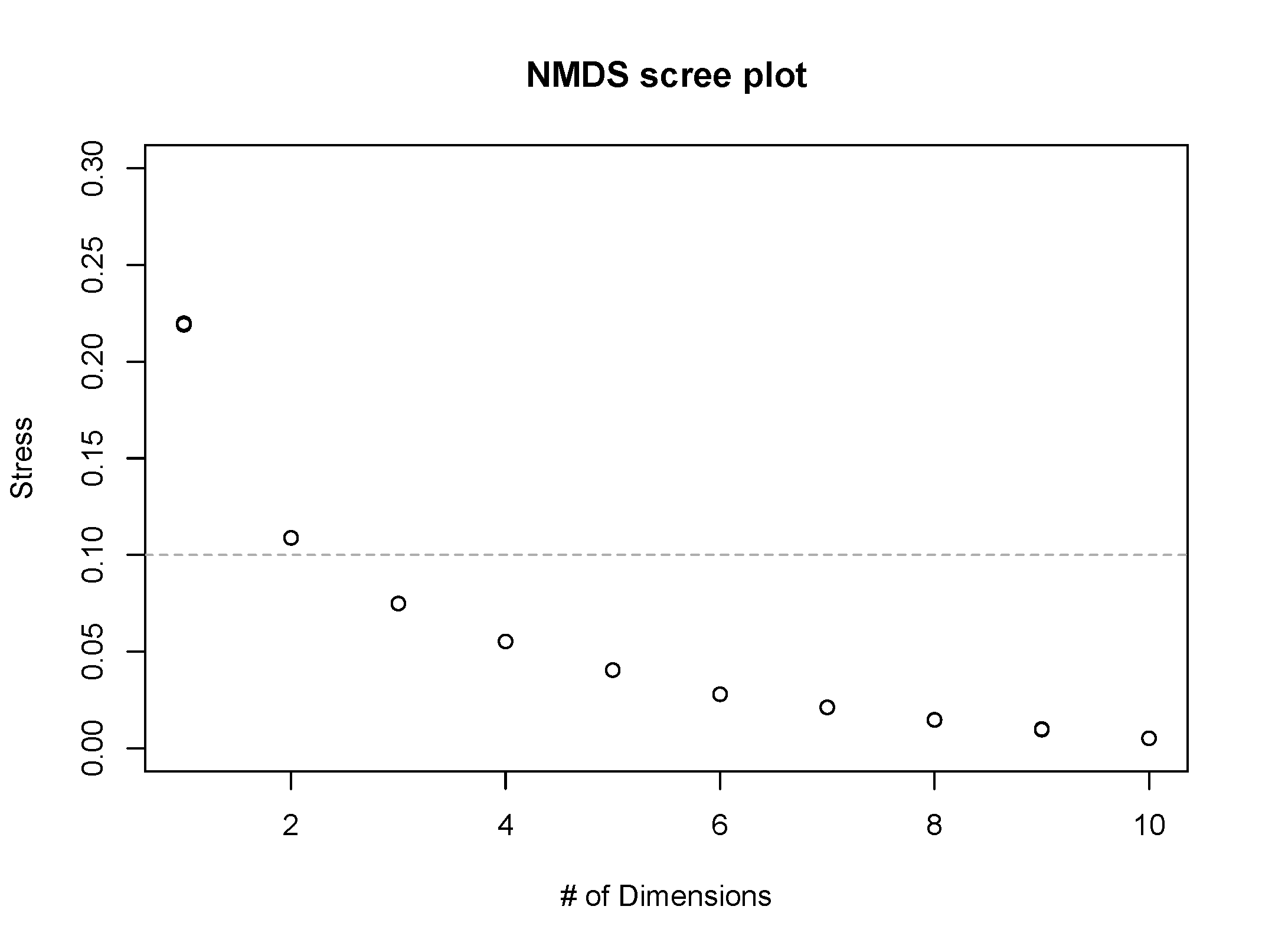
**

**Figure S2:** Scree plot of the Non-Metric Multidimensional Scaling (NMDS). Each point is the stress value of a single trial plotted against the number of dimensions. Ten trials were run on each dimension. The horizontal dashed line indicates the stress value under which a fit is considered good (0.10).

**
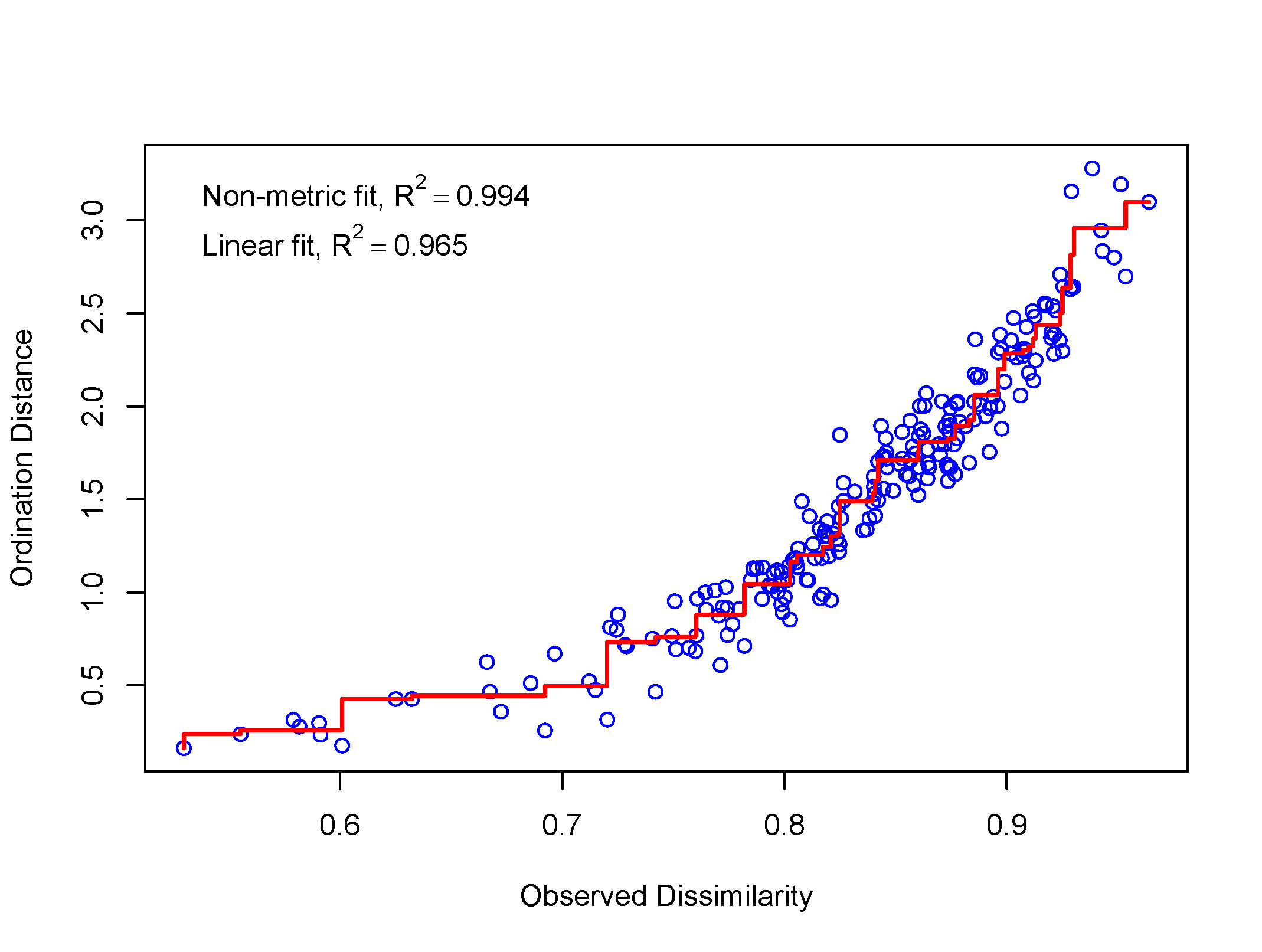
**

**Figure S3:** Stress plot of the Non-Metric Multidimensional Scaling (NMDS), showing Bray-Curtis dissimilarities between each pair of plots (Observed Dissimilarity) and the Euclidean distances between the same pair of plots in the 2-dimensional ordination space (Ordination Distance).


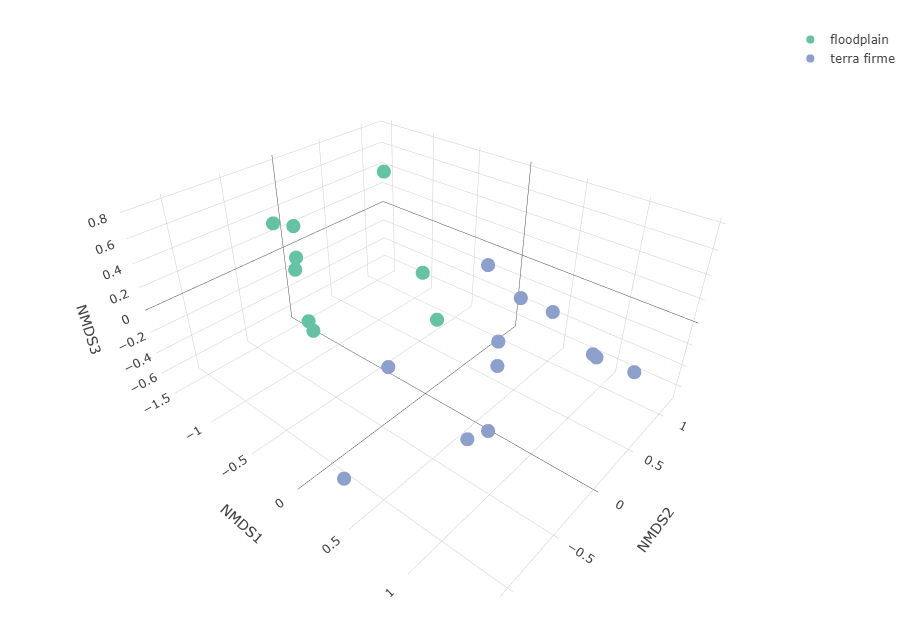


**Figure S4:** The NMDS ordination in three dimensions based on Bray-Curtis dissimilarity (stress value = 0.07). Green dots indicate floodplain plots while blue dots indicate terra firme plots.

**
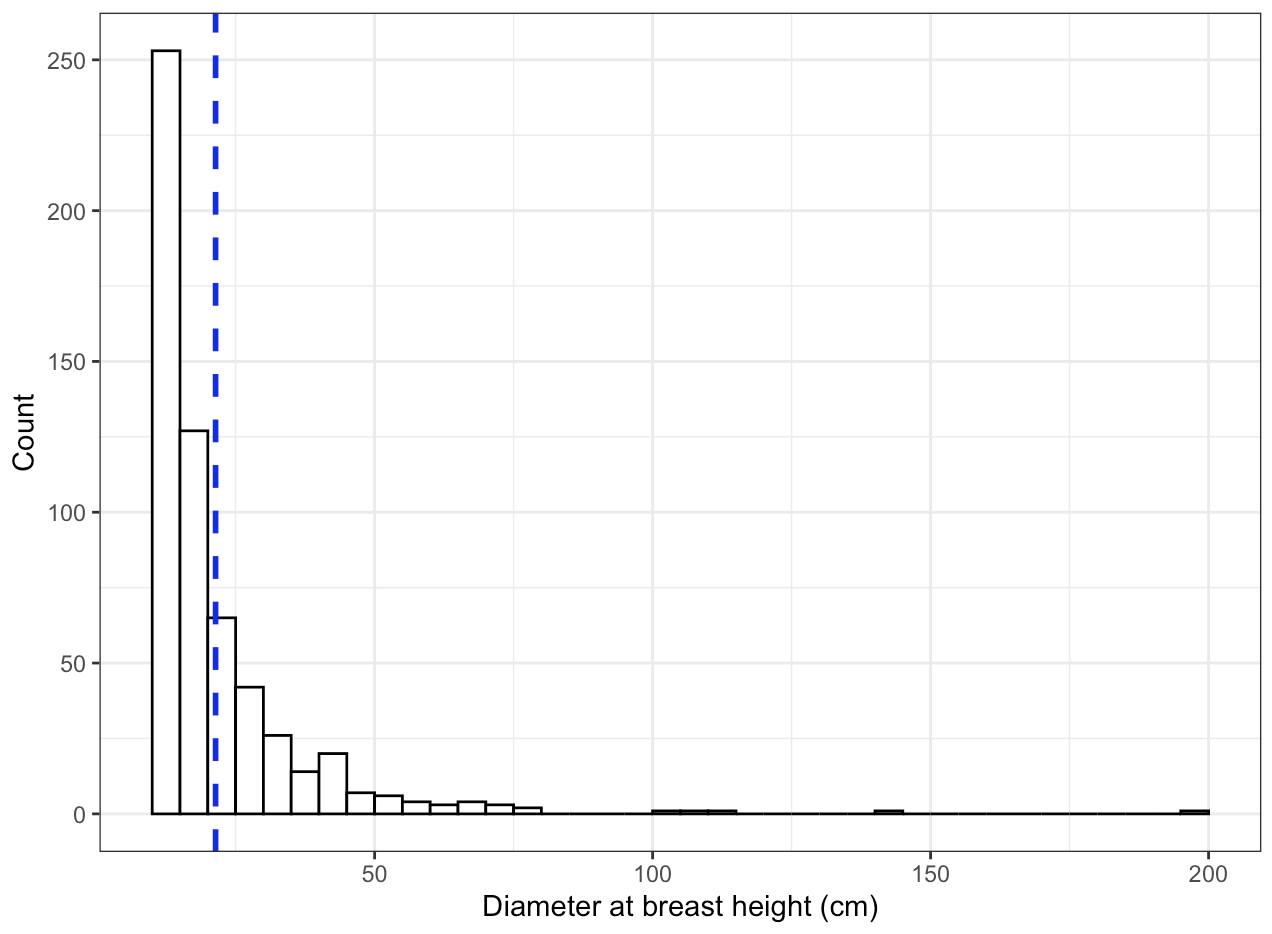
**

**Figure S5:** Histogram of tree diameters at breast height (DBH) in cm in FLP-01. The blue dashed line indicates the mean DBH of 21.4cm.


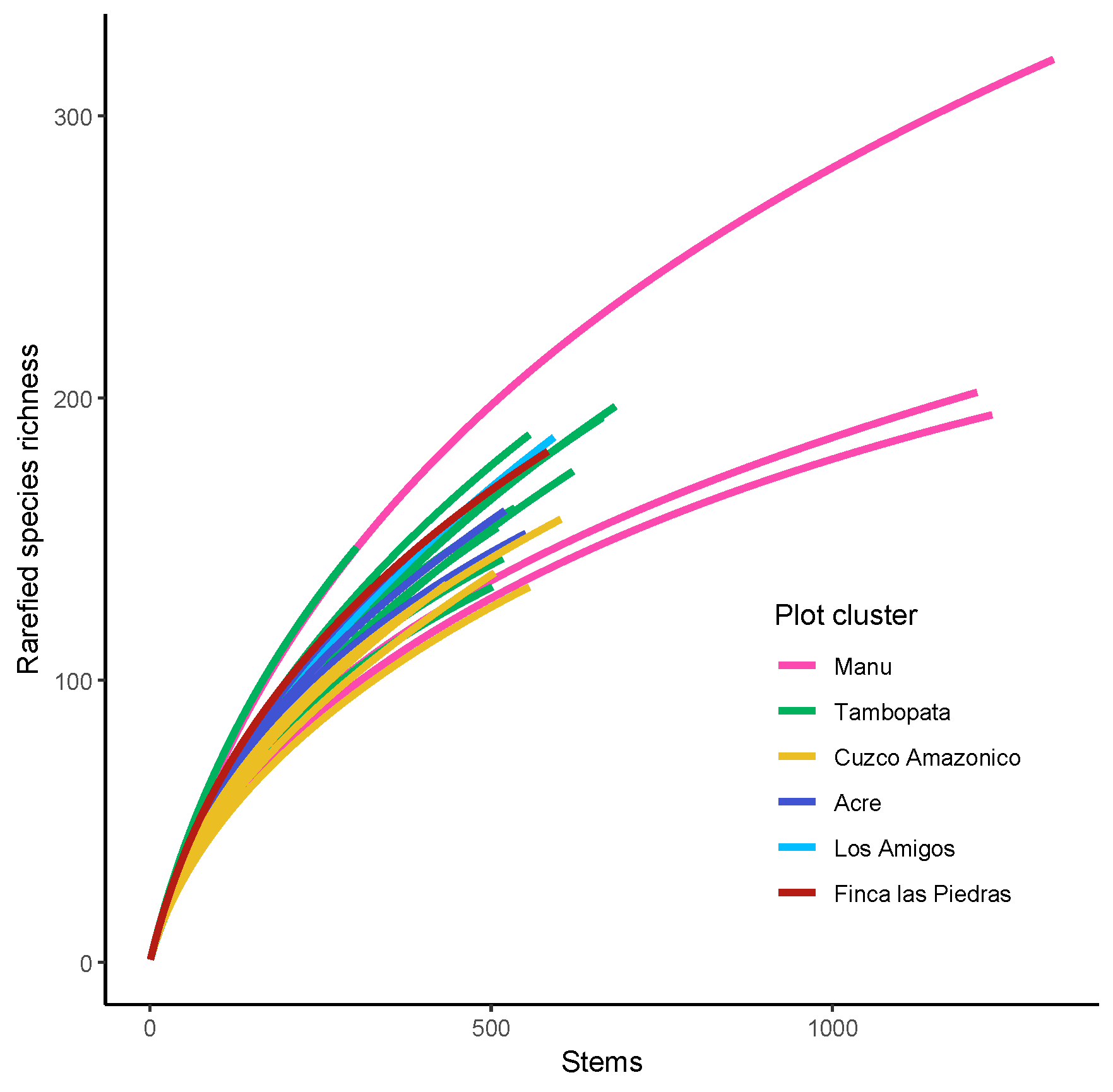


**Figure S6**: Rarefaction curves for each plot showing an increase in species richness based on increased sampling of stems ≥10 cm DBH. Colors represent the plot clusters as indicated in **Figs. 1 & 2**.


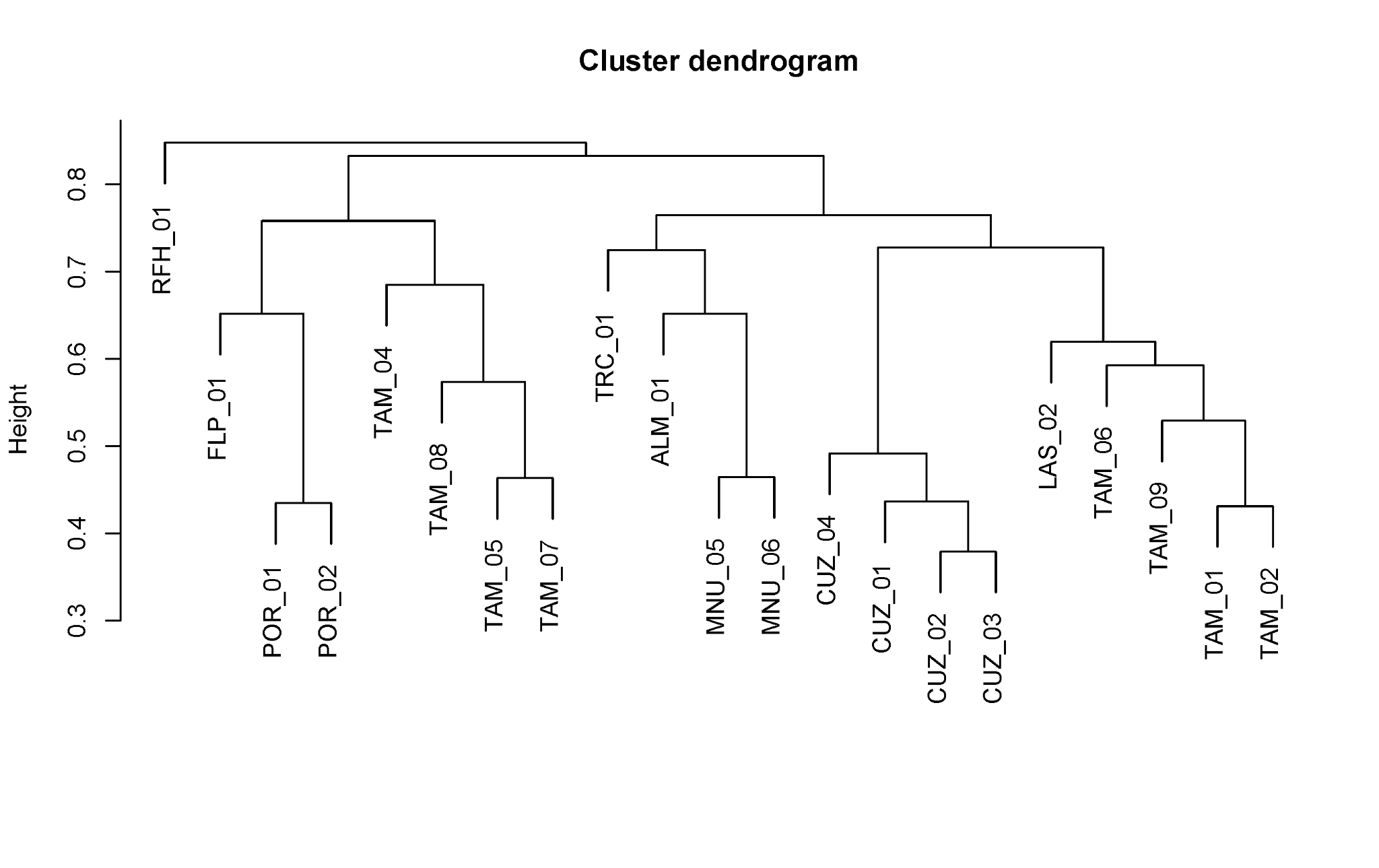


**Figure S7**: Cluster diagram of permanent forest dynamics plots based on Bray-Curtis dissimilarity.


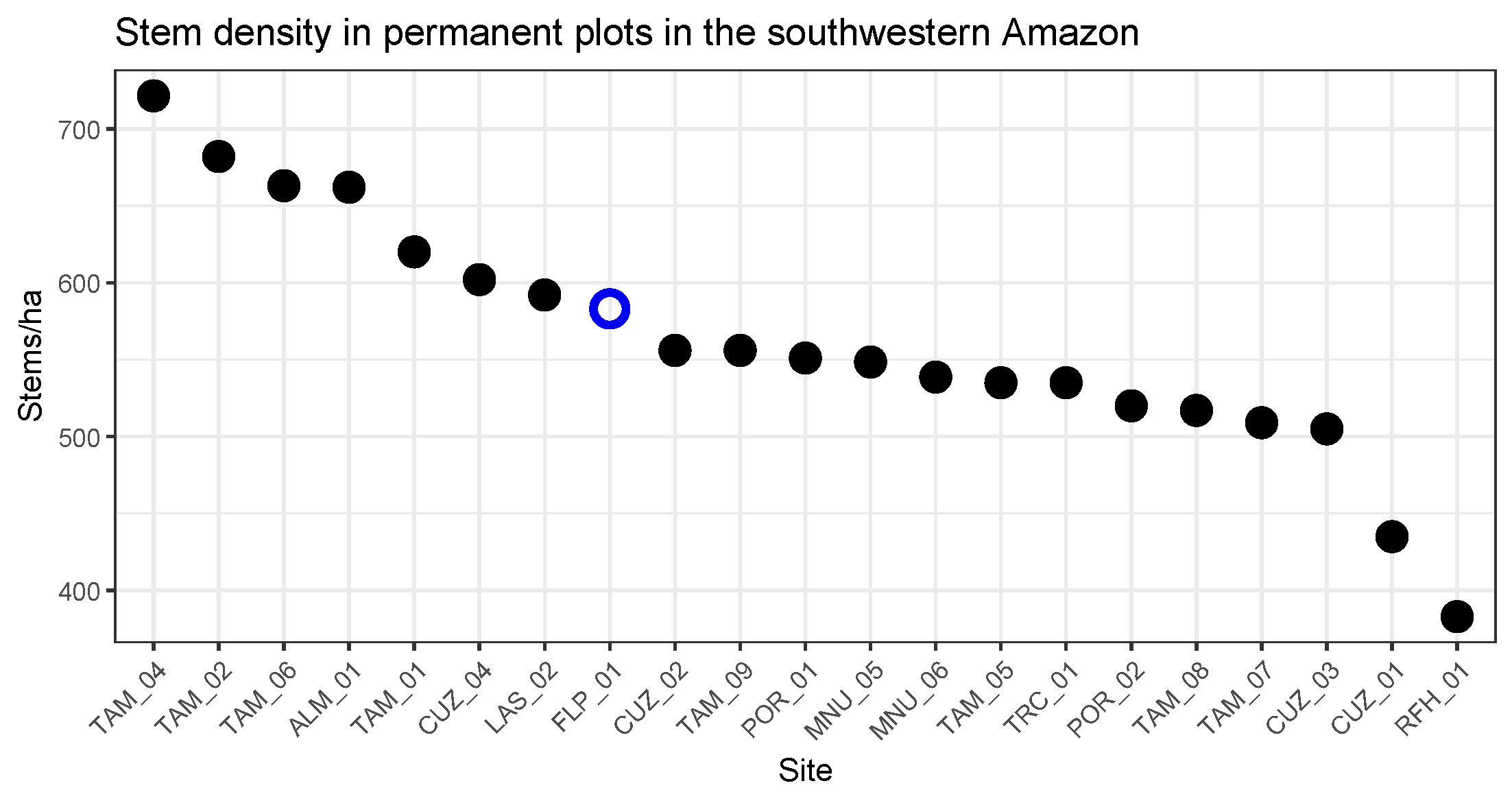


**Figure S8:** Stem densities per hectare of 21 plots in MDD, Peru and Acre, Brazil. FLP-01 is shown as an open blue circle. Note that plot data are from different years (see **Table 1**).

**
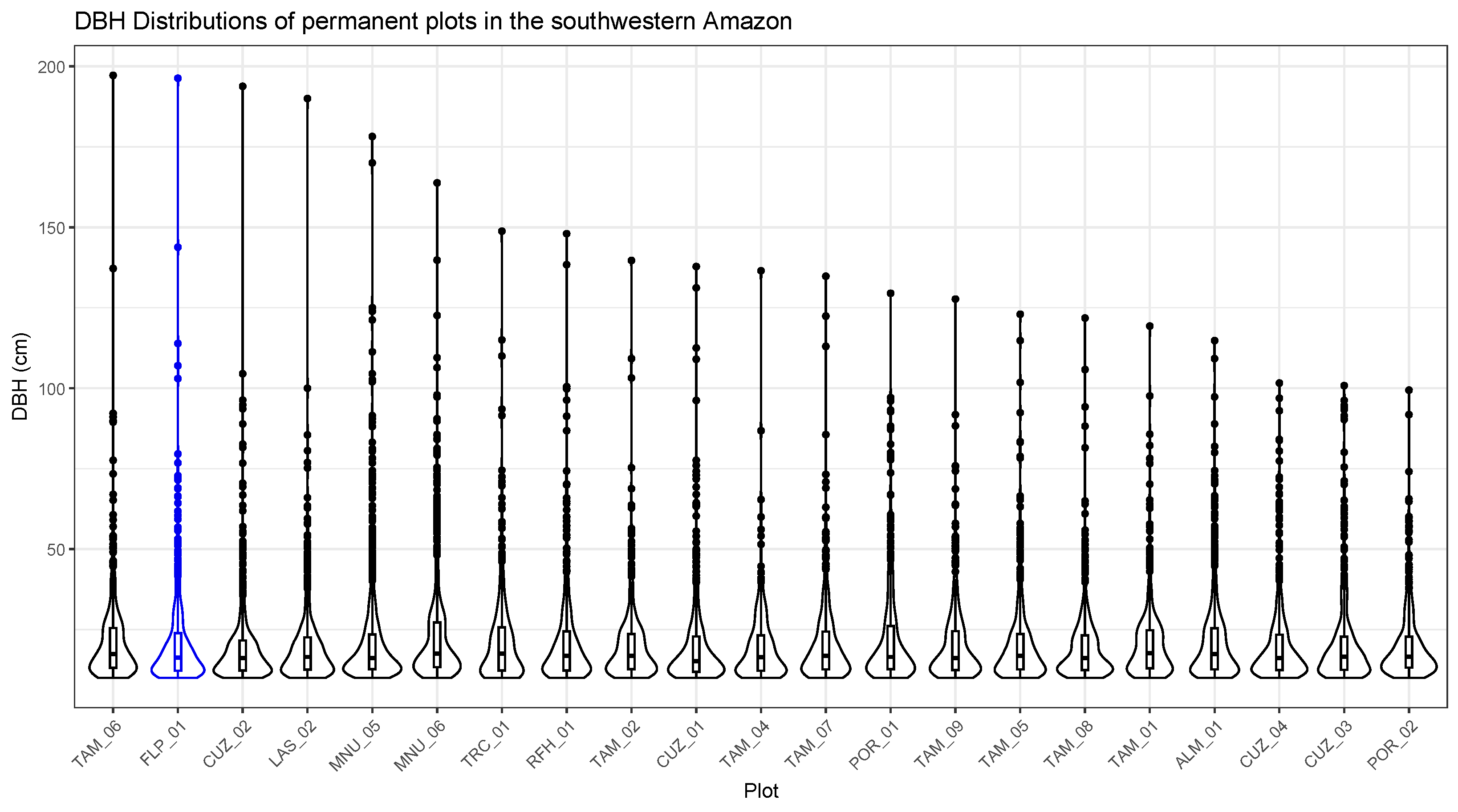
**

**Figure S9:** Violin plots showing DBH distributions of 21 plots in MDD, Peru and Acre, Brazil. The inner boxplots show the means and interquartile ranges. Dots represent outliers. Plots are arranged by descending maximum DBH, with FLP-01 shown in blue. Note that plot data are from different years (see **Table 1**).

**
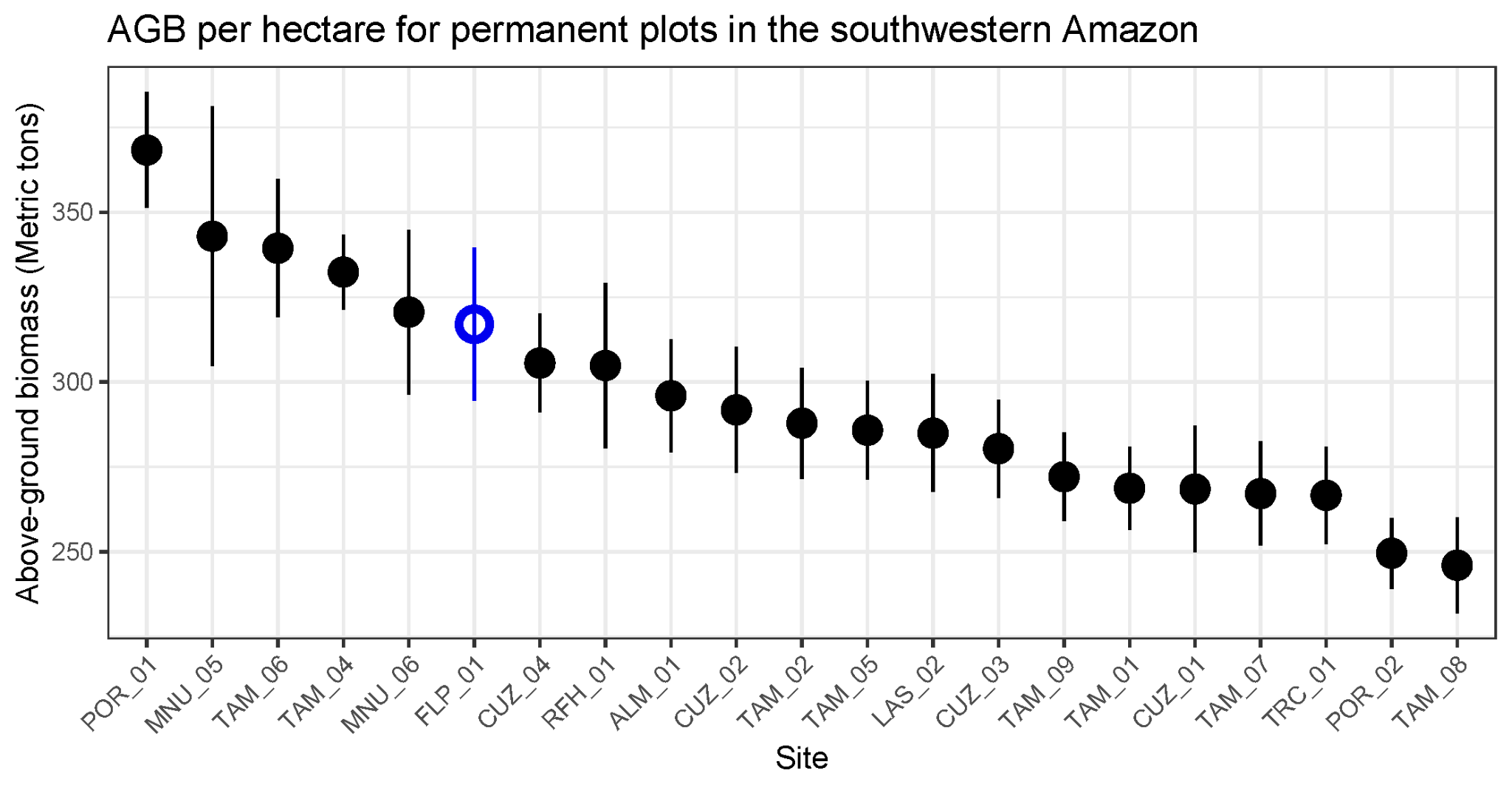
**

**Figure S10:** Aboveground biomass (AGB) per hectare of 21 plots in MDD, Peru and Acre, Brazil. The dots are AGB estimates while the whiskers are the standard deviations of the estimates. FLP-01 is shown as an open blue circle. Note that plot data are from different years (see **Table 1**).

**Table S1:** Table summarizing the number of individuals, maximum diameter at breast height in mm (max DBH), 95% quantile DBH, total basal area in cm2, and the percent of total plot basal area for each species (or morphospecies) in the Finca las Piedras permanent forest dynamics plot.

| **Family** | **Species** | **Number of individuals** | **Max DBH** | **95% quantile DBH** | **Total basal area** | **% of plot basal area** |
| --- | --- | --- | --- | --- | --- | --- |
| Anacardiaceae | *Tapirira guianensis* | 1 | 191 | 191 | 286.5 | 0.09 |
| Anacardiaceae | *Tapirira obtusa* | 1 | 115 | 115 | 103.9 | 0.03 |
| Annonaceae | *Oxandra mediocris* | 3 | 195 | 189.9 | 619.9 | 0.19 |
| Apocynaceae | *Aspidosperma rigidum* | 1 | 104 | 104 | 84.9 | 0.03 |
| Apocynaceae | *Aspidosperma tambopatense* | 8 | 290 | 288.6 | 2685.8 | 0.81 |
| Apocynaceae | *Geissospermum reticulatum* | 1 | 314 | 314 | 774.4 | 0.23 |
| Apocynaceae | *Tabernaemontana cymosa* | 1 | 177 | 177 | 246.1 | 0.07 |
| Araliaceae | *Schefflera morototoni* | 1 | 123 | 123 | 118.8 | 0.04 |
| Arecaceae | *Euterpe precatoria* | 28 | 187 | 186.3 | 5409.1 | 1.64 |
| Arecaceae | *Iriartea deltoidea* | 10 | 305 | 300.05 | 5328.6 | 1.61 |
| Bignoniaceae | *Jacaranda copaia* | 3 | 124 | 122.6 | 295.9 | 0.09 |
| Bixaceae | *Bixa excelsa* | 17 | 382 | 335.6 | 6954.8 | 2.11 |
| Boraginaceae | *Cordia ucayaliensis* | 4 | 179 | 178.55 | 671.7 | 0.20 |
| Burseraceae | *Crepidospermum goudotianum* | 1 | 153 | 153 | 183.9 | 0.06 |
| Burseraceae | *Protium amazonicum* | 2 | 113 | 112.75 | 191.9 | 0.06 |
| Burseraceae | *Protium aracouchini* | 1 | 108 | 108 | 91.6 | 0.03 |
| Burseraceae | *Protium sagotianum* | 2 | 392 | 387 | 1876.5 | 0.57 |
| Burseraceae | *Protium altissimum* | 14 | 728 | 656.5 | 21495.4 | 6.51 |
| Burseraceae | *Protium stevensonii* | 1 | 402 | 402 | 1269.2 | 0.38 |
| Cannabaceae | *Celtis schippii* | 2 | 130 | 128.9 | 224.3 | 0.07 |
| Caricaceae | *Jacaratia digitata* | 1 | 209 | 209 | 343.1 | 0.10 |
| Chrysobalanaceae | *Hirtella excelsa* | 1 | 449 | 449 | 1583.4 | 0.48 |
| Chrysobalanaceae | *Hirtella pilosissima* | 1 | 107 | 107 | 89.9 | 0.03 |
| Chrysobalanaceae | *Hirtella triandra* | 9 | 140 | 132.4 | 984.2 | 0.30 |
| Chrysobalanaceae | *Licania apetala* | 1 | 405 | 405 | 1288.2 | 0.39 |
| Chrysobalanaceae | *Licania canescens* | 2 | 160 | 157.75 | 304.9 | 0.09 |
| Chrysobalanaceae | *Licania caudata* | 1 | 153 | 153 | 183.9 | 0.06 |
| Clusiaceae | *Symphonia globulifera* | 10 | 413 | 400.4 | 6942.2 | 2.10 |
| Clusiaceae | *Tovomita sp1* | 1 | 204 | 204 | 326.9 | 0.10 |
| Clusiaceae | *Tovomita umbellata* | 1 | 105 | 105 | 86.6 | 0.03 |
| Dichapetalaceae | *Tapura juruana* | 1 | 204 | 204 | 326.9 | 0.10 |
| Elaeocarpaceae | *Sloanea eichleri* | 1 | 112 | 112 | 98.5 | 0.03 |
| Elaeocarpaceae | *Sloanea guianensis* | 6 | 187 | 177.25 | 880.3 | 0.27 |
| Euphorbiaceae | *Alchornea triplinervia* | 2 | 119 | 118.25 | 196.2 | 0.06 |
| Euphorbiaceae | *Aparisthmium cordatum* | 2 | 102 | 102 | 163.4 | 0.05 |
| Euphorbiaceae | *Conceveiba guianensis* | 9 | 310 | 258 | 2133.0 | 0.65 |
| Euphorbiaceae | *Pausandra trianae* | 20 | 173 | 172.05 | 2917.3 | 0.88 |
| Fabaceae | *Albizia niopoides* | 3 | 796 | 772.2 | 8389.5 | 2.54 |
| Fabaceae | *Dialium guianense* | 2 | 275 | 266.65 | 685.6 | 0.21 |
| Fabaceae | *Dussia tessmannii* | 1 | 233 | 233 | 426.4 | 0.13 |
| Fabaceae | *Inga acreana* | 1 | 202 | 202 | 320.5 | 0.10 |
| Fabaceae | *Inga alba* | 3 | 688 | 663.6 | 6586.2 | 2.00 |
| Fabaceae | *Inga auristellae* | 1 | 159 | 159 | 198.6 | 0.06 |
| Fabaceae | *Inga bourgoni* | 1 | 141 | 141 | 156.1 | 0.05 |
| Fabaceae | *Inga capitata* | 3 | 183 | 182.7 | 723.6 | 0.22 |
| Fabaceae | *Inga chartacea* | 1 | 262 | 262 | 539.1 | 0.16 |
| Fabaceae | *Inga cinnamomea* | 1 | 114 | 114 | 102.1 | 0.03 |
| Fabaceae | *Inga coruscans* | 1 | 114 | 114 | 102.1 | 0.03 |
| Fabaceae | *Inga laurina* | 3 | 511 | 486.2 | 2757.0 | 0.84 |
| Fabaceae | *Inga marginata* | 1 | 104 | 104 | 84.9 | 0.03 |
| Fabaceae | *Inga thibaudiana* | 4 | 187 | 178 | 571.2 | 0.17 |
| Fabaceae | *Poeppigia procera* | 1 | 144 | 144 | 162.9 | 0.05 |
| Fabaceae | *Pseudopiptadenia suaveolens* | 1 | 768 | 768 | 4632.5 | 1.40 |
| Fabaceae | *Tachigali alba* | 2 | 665 | 637.5 | 3577.1 | 1.08 |
| Fabaceae | *Tachigali poeppigiana* | 13 | 532 | 343.6 | 3973.9 | 1.20 |
| Humiriaceae | *Humiriastrum excelsum* | 1 | 357 | 357 | 1001.0 | 0.30 |
| Humiriaceae | *Sacoglottis mattogrossensis* | 1 | 217 | 217 | 369.8 | 0.11 |
| Hypericaceae | *Vismia macrophylla* | 1 | 117 | 117 | 107.5 | 0.03 |
| Icacinaceae | *Calatola costaricensis* | 6 | 212 | 206.5 | 1441.8 | 0.44 |
| Lauraceae | *Aniba muca* | 1 | 131 | 131 | 134.8 | 0.04 |
| Lauraceae | *Beilschmiedia tovarensis* | 1 | 568 | 568 | 2533.9 | 0.77 |
| Lauraceae | *Endlicheria formosa* | 1 | 125 | 125 | 122.7 | 0.04 |
| Lauraceae | *Endlicheria krukovii* | 1 | 138 | 198.1 | 149.6 | 0.05 |
| Lauraceae | *Endlicheria sp1* | 3 | 205 | 138 | 563.6 | 0.17 |
| Lauraceae | *Lauraceae sp1* | 3 | 417 | 404.4 | 2114.1 | 0.64 |
| Lauraceae | *Licaria aurea* | 1 | 239 | 239 | 448.6 | 0.14 |
| Lauraceae | *Nectandra cissiflora* | 1 | 445 | 445 | 1555.3 | 0.47 |
| Lauraceae | *Nectandra cuspidata* | 1 | 102 | 102 | 81.7 | 0.02 |
| Lauraceae | *Nectandra longifolia* | 2 | 300 | 186 | 1201.7 | 0.36 |
| Lauraceae | *Nectandra pulverulenta* | 1 | 222 | 297.55 | 387.1 | 0.12 |
| Lauraceae | *Nectandra sp1* | 1 | 186 | 222 | 271.7 | 0.08 |
| Lauraceae | *Ocotea bofo* | 1 | 566 | 566 | 2516.1 | 0.76 |
| Lauraceae | *Ocotea floribunda* | 1 | 141 | 141 | 156.1 | 0.05 |
| Lauraceae | *Ocotea oblonga* | 2 | 227 | 221.8 | 523.5 | 0.16 |
| Lauraceae | *Ocotea tessmannii* | 1 | 337 | 337 | 892.0 | 0.27 |
| Lauraceae | *Persea americana* | 6 | 247 | 226 | 1245.6 | 0.38 |
| Lauraceae | *Pleurothyrium vasquezii* | 1 | 189 | 189 | 280.6 | 0.08 |
| Lecythidaceae | *Bertholletia excelsa* | 3 | 1963 | 1880.6 | 40611.8 | 12.30 |
| Lecythidaceae | *Couratari macrosperma* | 1 | 719 | 719 | 4060.2 | 1.23 |
| Lecythidaceae | *Eschweilera coriacea* | 4 | 191 | 188.75 | 917.8 | 0.28 |
| Lecythidaceae | *Eschweilera gigantea* | 1 | 139 | 139 | 151.7 | 0.05 |
| Lecythidaceae | *Eschweilera tessmannii* | 2 | 496 | 486.4 | 2658.0 | 0.81 |
| Linaceae | *Roucheria columbiana* | 1 | 211 | 211 | 349.7 | 0.11 |
| Malvaceae | *Apeiba membranacea* | 10 | 466 | 429.1 | 5927.2 | 1.80 |
| Malvaceae | *Eriotheca globosa* | 1 | 108 | 108 | 91.6 | 0.03 |
| Malvaceae | *Luehea grandiflora* | 1 | 187 | 187 | 274.6 | 0.08 |
| Malvaceae | *Matisia ochrocalyx* | 2 | 171 | 170.95 | 456.6 | 0.14 |
| Malvaceae | *Sterculia peruviana* | 1 | 124 | 124 | 120.8 | 0.04 |
| Malvaceae | *Theobroma cacao* | 1 | 128 | 128 | 128.7 | 0.04 |
| Malvaceae | *Theobroma speciosum* | 1 | 145 | 145 | 165.1 | 0.05 |
| Meliaceae | *Cabralea canjerana* | 2 | 209 | 204.35 | 448.8 | 0.14 |
| Meliaceae | *Cedrela fissilis* | 2 | 327 | 322.95 | 1315.1 | 0.40 |
| Meliaceae | *Guarea kunthiana* | 5 | 593 | 215 | 4602.7 | 1.39 |
| Meliaceae | *Guarea pterorhachis* | 3 | 131 | 553.6 | 365.0 | 0.11 |
| Meliaceae | *Guarea sp1* | 1 | 215 | 130.4 | 363.1 | 0.11 |
| Meliaceae | *Trichilia quadrijuga* | 6 | 165 | 164.25 | 876.8 | 0.27 |
| Meliaceae | *Trichilia septentrionalis* | 1 | 108 | 108 | 91.6 | 0.03 |
| Monimiaceae | *Mollinedia killipii* | 1 | 134 | 134 | 141.0 | 0.04 |
| Monimiaceae | *Mollinedia ovata* | 3 | 131 | 128.6 | 311.3 | 0.09 |
| Moraceae | *Brosimum acutifolium* | 2 | 532 | 525.9 | 3543.1 | 1.07 |
| Moraceae | *Brosimum alicastrum* | 1 | 198 | 198 | 307.9 | 0.09 |
| Moraceae | *Brosimum guianense* | 2 | 273 | 265.3 | 696.6 | 0.21 |
| Moraceae | *Brosimum lactescens* | 1 | 114 | 114 | 102.1 | 0.03 |
| Moraceae | *Brosimum rubescens* | 1 | 125 | 125 | 122.7 | 0.04 |
| Moraceae | *Castilla ulei* | 3 | 318 | 303.5 | 1196.7 | 0.36 |
| Moraceae | *Ficus gomelleira* | 1 | 1438 | 1438 | 16240.8 | 4.92 |
| Moraceae | *Helicostylis tomentosa* | 2 | 265 | 259.35 | 733.0 | 0.22 |
| Moraceae | *Maquira calophylla* | 3 | 359 | 354.6 | 2470.4 | 0.75 |
| Moraceae | *Maquira coriacea* | 1 | 465 | 465 | 1698.2 | 0.51 |
| Moraceae | *Maquira guianensis* | 1 | 172 | 172 | 232.4 | 0.07 |
| Moraceae | *Naucleopsis krukovii* | 4 | 326 | 308.6 | 1478.5 | 0.45 |
| Moraceae | *Naucleopsis pseudonaga* | 1 | 196 | 196 | 301.7 | 0.09 |
| Moraceae | *Perebea angustifolia* | 2 | 204 | 200.65 | 474.3 | 0.14 |
| Moraceae | *Perebea tessmannii* | 1 | 288 | 288 | 651.4 | 0.20 |
| Moraceae | *Pseudolmedia laevigata* | 6 | 173 | 170 | 1028.6 | 0.31 |
| Moraceae | *Pseudolmedia laevis* | 7 | 515 | 476.9 | 5556.8 | 1.68 |
| Moraceae | *Pseudolmedia macrophylla* | 1 | 185 | 185 | 268.8 | 0.08 |
| Moraceae | *Pseudolmedia spuria* | 1 | 107 | 107 | 89.9 | 0.03 |
| Myristicaceae | *Iryanthera juruensis* | 9 | 225 | 219.8 | 1974.8 | 0.60 |
| Myristicaceae | *Iryanthera laevis* | 13 | 1070 | 672.8 | 14329.3 | 4.34 |
| Myristicaceae | *Iryanthera ulei* | 1 | 260 | 260 | 530.9 | 0.16 |
| Myristicaceae | *Otoba parvifolia* | 1 | 109 | 109 | 93.3 | 0.03 |
| Myristicaceae | *Virola flexuosa* | 1 | 100 | 100 | 78.5 | 0.02 |
| Myristicaceae | *Virola multinervia* | 1 | 138 | 138 | 149.6 | 0.05 |
| Myristicaceae | *Virola sebifera* | 1 | 320 | 320 | 804.2 | 0.24 |
| Myrtaceae | *Eugenia densiflora* | 2 | 424 | 410.95 | 1620.6 | 0.49 |
| Myrtaceae | *Eugenia florida* | 1 | 103 | 103 | 83.3 | 0.03 |
| Myrtaceae | *Eugenia sp1* | 1 | 206 | 206 | 333.3 | 0.10 |
| Myrtaceae | *Myrcia crebra* | 7 | 283 | 277.6 | 2956.1 | 0.90 |
| Myrtaceae | *Myrcia ruiziana* | 1 | 194 | 172.95 | 295.6 | 0.09 |
| Myrtaceae | *Myrcia sp1* | 2 | 175 | 194 | 381.6 | 0.12 |
| Nyctaginaceae | *Guapira myrtiflora* | 2 | 122 | 121 | 198.6 | 0.06 |
| Nyctaginaceae | *Guapira opposita* | 1 | 311 | 311 | 759.6 | 0.23 |
| Nyctaginaceae | *Neea sp1* | 1 | 422 | 422 | 1398.7 | 0.42 |
| Ochnaceae | *Ouratea discophora* | 2 | 110 | 109.9 | 186.6 | 0.06 |
| Ochnaceae | *Quiina florida* | 2 | 184 | 180.4 | 364.4 | 0.11 |
| Olacaceae | *Heisteria acuminata* | 1 | 109 | 109 | 93.3 | 0.03 |
| Olacaceae | *Minquartia guianensis* | 2 | 211 | 208.95 | 576.6 | 0.17 |
| Peraceae | *Chaetocarpus echinocarpus* | 1 | 324 | 324 | 824.5 | 0.25 |
| Phyllanthaceae | *Margaritaria nobilis* | 5 | 162 | 154.8 | 533.6 | 0.16 |
| Putranjivaceae | *Drypetes amazonica* | 2 | 298 | 289.15 | 812.5 | 0.25 |
| Putranjivaceae | *Drypetes gentryana* | 1 | 152 | 152 | 181.5 | 0.05 |
| Rhizophoraceae | *Cassipourea peruviana* | 7 | 179 | 175.7 | 1136.3 | 0.34 |
| Rubiaceae | *Alibertia isernii* | 1 | 131 | 131 | 134.8 | 0.04 |
| Rubiaceae | *Alseis labatioides* | 3 | 275 | 259.7 | 805.9 | 0.24 |
| Rubiaceae | *Amaioua guianensis* | 2 | 177 | 174.75 | 382.9 | 0.12 |
| Rubiaceae | *Capirona decorticans* | 4 | 321 | 316.8 | 2053.3 | 0.62 |
| Rubiaceae | *Faramea torquata* | 2 | 112 | 112 | 197.0 | 0.06 |
| Rubiaceae | *Posoqueria latifolia* | 2 | 116 | 115.55 | 195.6 | 0.06 |
| Rutaceae | *Galipea trifoliata* | 23 | 220 | 193.7 | 3677.9 | 1.11 |
| Rutaceae | *Metrodorea flavida* | 2 | 221 | 217.9 | 582.2 | 0.18 |
| Sabiaceae | *Meliosma herbertii* | 6 | 444 | 424.5 | 3642.6 | 1.10 |
| Salicaceae | *Casearia combaymensis* | 1 | 313 | 313 | 769.4 | 0.23 |
| Salicaceae | *Casearia javitensis* | 1 | 115 | 115 | 103.9 | 0.03 |
| Salicaceae | *Casearia obovalis* | 4 | 197 | 187.4 | 615.3 | 0.19 |
| Salicaceae | *Casearia sylvestris* | 4 | 193 | 191.65 | 1031.3 | 0.31 |
| Salicaceae | *Laetia procera* | 8 | 663 | 507.6 | 4556.8 | 1.38 |
| Sapindaceae | *Toulicia reticulata* | 1 | 122 | 122 | 116.9 | 0.04 |
| Sapotaceae | *Chrysophyllum lucentifolium* | 3 | 643 | 618.9 | 5101.8 | 1.55 |
| Sapotaceae | *Diploon cuspidatum* | 2 | 225 | 220.3 | 532.4 | 0.16 |
| Sapotaceae | *Manilkara bidentata* | 1 | 330 | 330 | 855.3 | 0.26 |
| Sapotaceae | *Micropholis egensis* | 2 | 1030 | 984.6 | 8449.2 | 2.56 |
| Sapotaceae | *Micropholis guianensis* | 5 | 715 | 661.2 | 7429.5 | 2.25 |
| Sapotaceae | *Micropholis venulosa* | 1 | 387 | 387 | 1176.3 | 0.36 |
| Sapotaceae | *Pouteria ephedrantha* | 1 | 489 | 489 | 1878.1 | 0.57 |
| Sapotaceae | *Pouteria franciscana* | 2 | 180 | 176.25 | 341.1 | 0.10 |
| Sapotaceae | *Pouteria macrophylla* | 1 | 102 | 102 | 81.7 | 0.02 |
| Siparunaceae | *Siparuna cuspidata* | 4 | 176 | 170.45 | 645.7 | 0.20 |
| Siparunaceae | *Siparuna decipiens* | 29 | 223 | 215 | 5157.9 | 1.56 |
| Siparunaceae | *Siparuna guianensis* | 1 | 112 | 112 | 98.5 | 0.03 |
| Staphyleaceae | *Turpinia occidentalis* | 1 | 110 | 110 | 95.0 | 0.03 |
| Ulmaceae | *Ampelocera edentula* | 1 | 116 | 116 | 105.7 | 0.03 |
| Ulmaceae | *Ampelocera ruizii* | 1 | 250 | 250 | 490.9 | 0.15 |
| Urticaceae | *Cecropia sciadophylla* | 15 | 470 | 453.2 | 10758.3 | 3.26 |
| Urticaceae | *Pourouma cuspidata* | 6 | 320 | 280 | 1447.7 | 0.44 |
| Urticaceae | *Pourouma minor* | 11 | 381 | 365 | 5480.0 | 1.66 |
| Violaceae | *Leonia glycycarpa* | 7 | 215 | 214.7 | 1809.3 | 0.55 |
| Violaceae | *Rinorea viridiflora* | 2 | 108 | 107.8 | 176.6 | 0.05 |
| Violaceae | *Rinoreocarpus ulei* | 6 | 243 | 234.5 | 1761.7 | 0.53 |
| Vochysiaceae | *Qualea grandiflora* | 2 | 690 | 677.05 | 5198.2 | 1.57 |

**Table S2:** Table listing all significant indicator species in floodplain and terra firme forest. Listed are each species’ association values on a scale from 0-1 and their p-values.

| **Habitat** | | |
| --- | --- | --- |
| *Species* | Association value | P-value |
|  |  |  |
| **Floodplain** |  |  |
| *Brosimum alicastrum* | 0.891 | 0.01 |
| *Astrocaryum murumuru* | 0.827 | 0.02 |
| *Poulsenia armata* | 0.815 | 0.015 |
| *Lunania parviflora* | 0.812 | 0.005 |
| *Pseudomalmea diclina* | 0.796 | 0.01 |
| *Unonopsis matthewsii* | 0.784 | 0.05 |
| *Allophylus loretensis* | 0.745 | 0.02 |
| *Protium sagotianum* | 0.745 | 0.02 |
| *Pouteria franciscana* | 0.735 | 0.02 |
| *Trichilia adolfi* | 0.734 | 0.02 |
| *Attalea cephalotes* | 0.733 | 0.04 |
| *Micropholis egensis* | 0.721 | 0.03 |
| *Gustavia hexapetala* | 0.667 | 0.03 |
| *Perebea tessmannii* | 0.667 | 0.04 |
| *Sloanea latifolia* | 0.667 | 0.03 |
| *Swartzia myrtifolia* | 0.667 | 0.015 |
| *Trichilia elegans* | 0.667 | 0.02 |
| *Virola peruviana* | 0.667 | 0.03 |
| *Aniba terminalis* | 0.577 | 0.045 |
| *Calatola costaricensis* | 0.577 | 0.045 |
| *Inga quaternata* | 0.577 | 0.045 |
| *Oxandra mediocris* | 0.577 | 0.03 |
|  |  |  |
| **Terra firme** | | |
| *Pouteria indet* | 0.889 | 0.005 |
| *Inga indet* | 0.884 | 0.01 |
| *Oenocarpus bataua* | 0.872 | 0.015 |
| *Glycydendron amazonicum* | 0.864 | 0.005 |
| *Pseudolmedia macrophylla* | 0.841 | 0.02 |
| *Pourouma guianensis* | 0.824 | 0.05 |
| *Lacistema aggregatum* | 0.778 | 0.02 |
| *Apuleia leiocarpa* | 0.764 | 0.02 |
| *Sterculia indet* | 0.764 | 0.01 |
| *Aiouea grandifolia* | 0.707 | 0.04 |
| *Eschweilera indet* | 0.707 | 0.04 |
| *Licania indet* | 0.707 | 0.045 |
| *Naucleopsis ternstroemiiflora* | 0.707 | 0.04 |
| *Guatteria alutacea* | 0.645 | 0.04 |
| *Pouteria tarapotensis* | 0.645 | 0.045 |
| *Talisia indet* | 0.645 | 0.045 |
| *Virola indet* | 0.645 | 0.04 |

**Table S3:** Table showing the ten species with highest indicator value index (IVI) scores for floodplain and terra firme plots. Also shown are each species’ total basal area (BA) in m^2^, BA per hectare in m^2^, relative BA expressed as a percentage, total number of individuals (count), number of individuals per ha, and relative density expressed as a percentage.

| **Habitat** | | | | | | | |
| --- | --- | --- | --- | --- | --- | --- | --- |
| *Species* | **IVI** | **Total BA** | **BA ha^-1^** | **Rel. BA** | **Count** | **Count ha^-1^** | **Rel. Density** |
| **Floodplain** | | | | | | | |
| *Iriartea deltoidea* | 8.26 | 25.77 | 2.24 | 7.64 | 559 | 48.6 | 8.87 |
| *Pseudolmedia laevis* | 5.16 | 17.17 | 1.49 | 5.09 | 329 | 28.6 | 5.22 |
| *Otoba parvifolia* | 4.15 | 12.23 | 1.06 | 3.63 | 294 | 25.6 | 4.67 |
| *Quararibea wittii* | 2.94 | 6.89 | 0.60 | 2.04 | 242 | 21.0 | 3.84 |
| *Poulsenia armata* | 1.73 | 8.43 | 0.73 | 2.50 | 61 | 5.3 | 0.97 |
| *Pouteria ephedrantha* | 1.62 | 5.98 | 0.52 | 1.77 | 92 | 8.0 | 1.46 |
| *Astrocaryum murumuru* | 1.58 | 2.81 | 0.24 | 0.83 | 147 | 12.8 | 2.33 |
| *Scheelea cephalotes* | 1.57 | 5.97 | 0.52 | 1.77 | 86 | 7.5 | 1.36 |
| *Theobroma cacao* | 1.51 | 2.74 | 0.24 | 0.81 | 139 | 12.1 | 2.21 |
| *Attalea cephalotes* | 1.39 | 5.51 | 0.48 | 1.64 | 72 | 6.3 | 1.14 |
|  | | | | | | | |
| **Terra firme** | | | | | | | |
| *Iriartea deltoidea* | 7.12 | 22.80 | 1.70 | 6.70 | 534 | 39.8 | 7.54 |
| *Pourouma minor* | 2.69 | 9.06 | 0.68 | 2.66 | 193 | 14.4 | 2.72 |
| *Protium altissimum* | 2.31 | 8.93 | 0.67 | 2.62 | 142 | 10.6 | 2.00 |
| *Euterpe precatoria* | 2.25 | 4.04 | 0.30 | 1.19 | 234 | 17.4 | 3.30 |
| *Bertholletia excelsa* | 2.17 | 13.97 | 1.04 | 4.10 | 17 | 1.3 | 0.24 |
| *Pseudolmedia laevis* | 2.14 | 7.15 | 0.53 | 2.10 | 155 | 11.5 | 2.19 |
| *Leonia glycycarpa* | 1.85 | 4.98 | 0.37 | 1.46 | 158 | 11.8 | 2.23 |
| *Siparuna decipiens* | 1.47 | 2.39 | 0.18 | 0.70 | 159 | 11.8 | 2.24 |
| *Iryanthera juruensis* | 1.45 | 2.50 | 0.19 | 0.74 | 154 | 11.5 | 2.17 |
| *Iryanthera laevis* | 1.05 | 3.29 | 0.25 | 0.97 | 80 | 6.0 | 1.13 |
